# Spiralyde A, an Antikinetoplastid Dolabellane from the Brown Alga *Dictyota spiralis*

**DOI:** 10.3390/md17030192

**Published:** 2019-03-25

**Authors:** Olfa Chiboub, Ines Sifaoui, Jacob Lorenzo-Morales, Manef Abderrabba, Mondher Mejri, José Javier Fernández, José E. Piñero, Ana R. Díaz-Marrero

**Affiliations:** 1 Instituto Universitario de Bio-Orgánica Antonio González (IUBO AG), Centro de Investigaciones Biomédicas de Canarias (CIBICAN), Universidad de La Laguna (ULL), Avda. Astrofísico F. Sánchez, 2, 38206 La Laguna, Tenerife, Spain; alu0101168852@ull.edu.es; 2 Instituto Universitario de Enfermedades Tropicales y Salud Pública de Islas Canarias, Universidad de La Laguna, Avda. Astrofísico F. Sánchez s/n, 38206 La Laguna, Tenerife, Spain; isifaoui@ull.edu.es (I.S.); jmlorenz@ull.edu.es (J.L.-M.); jpinero@ull.edu.es (J.E.P.); 3 Laboratoire Matériaux-Molécules et Applications, IPEST, B.P 51, La Marsa, University of Carthage, Tunis 2070, Tunisia; manef.abderrabba@ipest.rnu.tn (M.A.); mondhermejri54@gmail.com (M.M.); 4 Institut National d’Agronomie de Tunis, 43 Avenue Charles Nicolle, University of Carthage, Tunis 1082, Tunisia; 5 Departamento de Química Orgánica, Universidad de La Laguna (ULL), Avda. Astrofísico F. Sánchez, 2, 38206 La Laguna, Tenerife, Spain

**Keywords:** Spiralyde A, brown algae, antikinetoplastid, dolabellanes, *Dictyota spiralis*, *Leishmania*, *Trypanosoma*

## Abstract

Bioassay-guided fractionation of the antikinetoplastid extract of the brown alga *Dictyota spiralis* has led to the isolation of spiralyde A (**1**), a new dolabellane aldehyde, along with other five known related diterpenes (**2**–**6**). Their structures were determined by HRESIMS, 1D and 2D NMR spectroscopy, and comparison with data reported in the literature. The antiparasitic activity of all compounds was evaluated. Spiralyde A (**1**) and the known compound 3,4-epoxy-7,18-dolabelladiene (**2**) were the most active compounds against *Leishmania amazonensis* and *Trypanosoma cruzi*. Spiralyde A (**1**) was the most potent compound, comparable to benznidazole, the reference drug for trypanocidal activity.

## 1. Introduction

Infections caused by kinetoplastid parasites, *Trypanosoma brucei* (human African trypanosomiasis, also, sleeping sickness), *Trypanosoma cruzi* (Chagas disease), and *Leishmania* spp. (leishmaniasis) are considered neglected tropical diseases (NTD) by the World Health Organization. Occurrence of drug resistance, toxicity and the lack of effective chemotherapy for the treatment of leishmaniasis and trypanosomiasis, urge a wide investigation to access new chemical entities with therapeutic potential [1].

Natural products are an important source of chemotherapeutic agents, in particular those used to treat infectious diseases. Marine natural products have provided the pharmaceutical industry with many potent compounds [2]. However, despite numerous marine molecules that have been tested to date in vitro for their trypanocidal and leishmanicidal activity, mainly obtained from sponges and corals [3,4,5], none has reached the market for the treatment of NTDs caused by kinetopastid parasites. 

In this context, seaweeds are attractive chemical starting points for drug discovery. As a country with 1148 km of coastline on the Mediterranean Sea, Tunisia is a rich source of biodiversity in terms of marine organisms. The city of Tabarka is located about 36°57′16′′ N 8°45′29′′ E, on the border with Algeria and is well known for coral fishing and diving. The 10-km long coastline of Tabarka on the northwestern Mediterranean coast of Tunisia is characterized by the presence of embayments, long extensions, headlands, pocket beaches, sand dunes and sea-cliffs. This characteristic allows a wide biodiversity, considering the various biotopes available [6].

In a previous study, a series of organic extracts of seven different species of Tunisian seaweeds were screened for such antioxidant and antiprotozoal activities, revealing the potential antiparasitic properties of some algal species [7]. As part of ongoing research aimed to find new antiparasitic agents from marine sources [8], the crude extract of the brown alga *Dictyota spiralis* showed the highest leishmanicidal and trypanocidal capacity.

**Figure 1:**
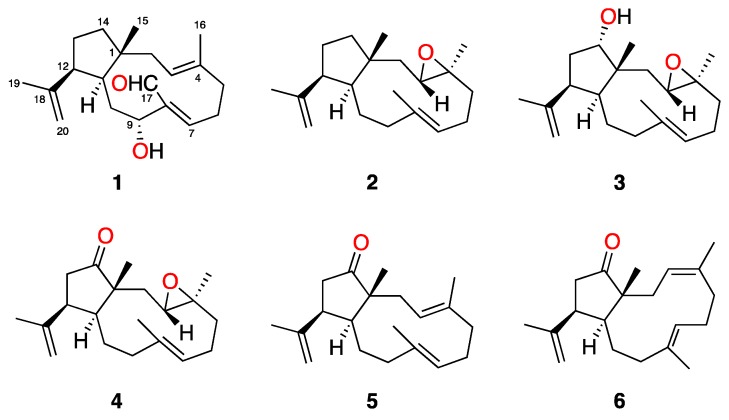
Structures of **1**–**6** isolated from *Dictyota spiralis*.

In order to find out the substances responsible for the antikinetoplastid activity, we carried out a bioassay-guided study on the extract of *Dictyota spiralis*, which led to the isolation of a new compound, spiralyde A (**1**), and five known dolabellane diterpenes (Figure 1). Their structures were elucidated on the basis of extensive spectroscopic analysis. Herein, we report the isolation and structure elucidation of these compounds, as well as their antikinetoplastid activity.

## 2. Results

### 2.1. Bioassay-Guided Isolation and Identification of Dolabellane Diterpenes

*Dictyota spiralis* was collected off the intertidal zone of the Northwest coast of Tunisia. Clean and dried specimens were powdered and extracted with dichloromethane (DCM) to afford an active crude extract against *Leishmania amazonensis* and *Trypanosoma cruzi* (Table 1). Gel filtration chromatography of 5 g of the extract afforded two active fractions, F3 (1.13 g) and F5 (283 mg). After sequential bioassay-guided fractionation and purification of both fractions, six dolabellane diterpenes, a previously unreported aldehyde derivative, spiralyde A (**1**), and five known compounds (**2**–**6**), were obtained (Figure 1). Their structures were determined on the basis of extensive spectroscopic analysis and comparison with data reported in the literature.

Compound **1** was obtained as an optically active, ${\left[\alpha \right]}_{D}^{20}$ = +21 (*c* 0.05, CH_2_Cl_2_), colorless oil. Its molecular formula C_20_H_30_O_2_ was deduced from the sodium adduct [M + Na]^+^ observed in the HRESIMS and indicated six degrees of unsaturation. The ^1^H NMR spectrum of **1** resembled those of **2**–**6** with some differences: the absence of one of the four characteristic methyl groups in a dolabellane skeleton and a deshielded signal at δ_H_ 10.02 (*J* = 2.0 Hz, 1H) (Table 2). These changes were attributed to the oxidation of one methyl group to aldehyde, also confirmed by the presence of a signal at δ_C_ 191.8 ppm in the ^13^C NMR and HSQC (Heteronuclear Single-Quantum Correlation) spectra. 

**Figure 2:**
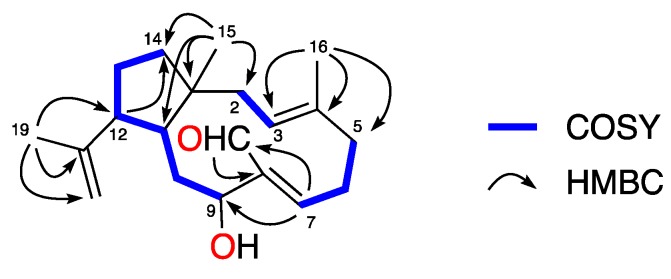
Selected COSY and key-HMBC correlations of **1**.

The full planar structure of **1** was assigned based on its 1D and 2D NMR spectroscopic data. The ^1^H-^1^H COSY (Correlation SpectroscopY) spectrum of **1** displayed a series of correlations establishing the presence of three spin systems: H_2_-2–H-3, H_2_-5–H-7, and H-9–H_2_-14 (Figure 2). Key HMBC (Heteronuclear Multiple Bond Correlation) correlations observed from H_3_-15 (δ_H_ 1.11) to C-1 (δ_C_ 47.1), C-2 (δ_C_ 42.3), C-11 (δ_C_ 42.1), and C-14 (δ_C_ 41.5); from H_3_-16 (δ_H_ 1.55) to C-3 (δ_C_ 127.4), C-4 (δ_C_ 134.1), and C-5 (δ_C_ 39.2); and correlations from H-7 (δ_H_ 6.31) to C-9 (δ_C_ 76.9) and C-17 (δ_C_ 191.8) and that of H-17 (δ_H_ 10.02) to C-8 (δ_C_ 139.3) permitted connection of the dolabellane carbon skeleton and positioned the aldehyde function at C-17. Additionally, the HMBC correlations observed from H_3_-19 (δ_H_ 1.53) to C-12 (δ_C_ 51.4), C-18 (δ_C_ 145.6), and C-20 (δ_C_ 111.4), confirmed an isoprenyl group attached to C-12. 

**Figure 3:**
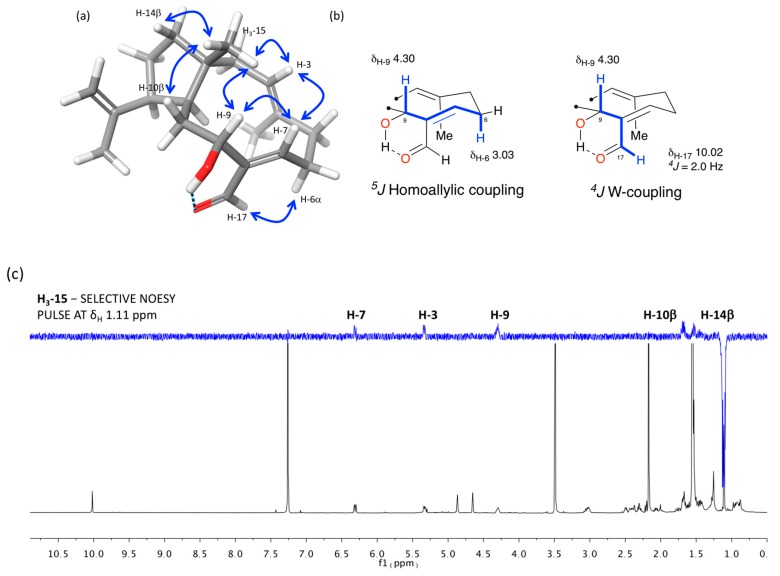
Relative configuration analysis: (**a**) key-NOESY correlations, (**b**) long-range COSY couplings, and (c) 1D-NOE experiment of **1**.

The relative configuration of the stereogenic centers and the geometries of the double bonds of **1** were assigned on the basis of 1D-selective NOESY and 2D ROESY experiments, long-range COSY correlations, and analysis of key ^1^H and ^13^C NMR data (Figure 3). NOE correlations observed from H_3_-15 to H-3, H-7, H-9, and the diastereotopic protons H-10β and H-14β located all these protons on the same face of the molecule. A NOESY correlation observed from H-12 to H-11 and the ^13^C chemical shift of C-19 at δ_C_ 23.1 confirmed the *trans*-fusion of the two rings and situated the isopropenyl group as β-oriented [9]. The *E* geometry of the Δ^3^ double bond was determined on the basis of the NOE correlation from H-3 to H-5β (δ_H_ 2.30), which is supported by a low chemical shift value of C-16 in the ^13^C NMR spectrum (δ_C_ 15.8). On the other hand, the NOESY correlations observed from H-7 to H-9, from H-17 to H-6α, as well as the long-range couplings observed in the COSY spectrum from H-9 to H-6α (δ_H_ 3.03) and H-17, respectively, probably favored by formation of an intramolecular hydrogen bond between the 9-OH and the C-17 carbonyl group, established a *Z* geometry for the Δ^7^ double bond. Therefore, the relative configuration of **1**, for which we propose the name of spiralyde A, is established as 1R*,3*E*,7*Z*,9R*,11S*,12S*.

The previously reported dolabellanes **2**–**6** were also isolated from the active fractions of the extract of *Dictyota spiralis*. Their structures were confirmed by comparison of their ^1^H and ^13^C NMR data with those described in the literature [9,10,11]. Compounds **2**–**4** were first isolated from specimens of *Dictyota dichotoma* collected in Italy [10]. Later, the revised structures of **2**, **4** and the 14-acetyl derivative of **3** were reported together with the isolation and structure elucidation of the stereoisomers **5** and **6** from an extract of *Dilophus spiralis* [9]. The analysis of the chemical shift of C-19 in the ^13^C NMR spectra of **2**–**6** was consistent with a β-oriented isopropenyl group at C-12, as shown in Figure 1. 

**Figure 4:**
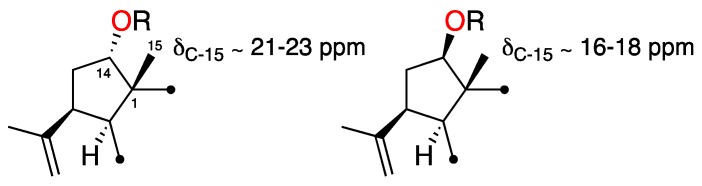
Relative configuration of C-1 and C-14 in 14-*O*-substituted dolabellanes (R = H or Ac).

3,4-Epoxy-14α-hydroxy-7,18-dolabelladiene (**3**), isolated as a yellowish oil, showed spectroscopic and physical properties in accordance with those previously described [10,11]. Its structure and relative configuration were confirmed based on the revised structure of the 14-acetyl derivative of **3**, also reported by Amico et al. [10,11]. Additionally, comparison of the ^13^C NMR data of **3** with those of other published 14-*O*-substituted dolabellanes [9,12], allowed us to conclude that the orientation of H-14 relative to Me-15 determines the chemical shift of C-15. As summarized in Figure 4, when H-14 and Me-15 are *cis*, the chemical shift value of C-15 is ca. δ_C-15_ ≈ 21.0–23.0 ppm, as is the case of **3**; whereas values of δ_C-15_ ≈ 16.0–18.0 indicate a *trans*-relationship. The presence of an acetyl or a hydroxyl group at C-14 does not influence the chemical shift value of C-15.

The absolute configurations of diterpenes **2**, the 14-acetyl derivative of **3**, **4–6** were reported by Ioannou et al. after single-crystal X-ray diffraction analysis and modified Mosher’s methods of two natural dolabellanes of the series [9]. Since we have confirmed that all spectroscopic and physical data of **2**–**6** agree with those previously reported, the same sign of the optical rotation of each compound corroborated the same absolute configuration. In addition, if we consider that co-occurring dolabellanes **1**–**6** isolated from *Dilophus spiralis* are the result of a common biosynthetic process, we could also propose the absolute configuration of spyralyde A (**1**) as 1*R*,3*E*,7*Z*,9*R*,11*S*,12*S*.

### 2.2. Antikinetoplastid Activity of Dolabellanes ***1***–***6***

The dichloromethane extract of *D. spiralis* exhibited a promising antikinetoplastid capacity with an IC_50_ of 9.76 ± 0.55 and 8.82 ± 0.98 µg/mL against the promastigote form of *L. amazonensis* and the epimastigote form of *T. cruzi,* respectively. Bioassay-guided fractionation of the crude extract yielded six dolabellanes **1**–**6**. Their in vitro antiprotozoal activity was evaluated applying serial dilutions of the compounds, and IC_50_ were calculated using the Alamar Blue reagent reaction. The obtained values of concentrations inhibiting 50% of parasites are summarized in Table 3 and expressed in µM. 

Both leishmanicidal and trypanocidal activities are based on a dose-dependent application for the active compounds **1** and **2**, meanwhile dolabellanes **3**–**6** did not show any activity below concentrations of 100 μM. Spiralyde A (**1**) showed the lowest IC_50_ (5.62 µM) value, comparable to the reference drug for trypanocidal treatment, benznidazole (6.95 µM). On the other side, **1** is more toxic than benznidazole when evaluated at concentration that inhibits 50% of murine macrophages.

## 3. Discussion

Identification of new molecules to treat kinetoplastid infections is an urgent need, and many efforts are focused on the search of natural products as potential sources of new chemical entities with antiprotozoal activities, both from terrestrial and marine origins [3,13,14,15].

Dolabellanes are metabolites commonly found in liverworts and marine organisms, mainly coelenterates (soft corals and gorgonians) and brown algae, including *Dictyota* species, but also opistobranch molluscs [16]. Some diterpenes of this family have proved to possess antiprotozoal capacity against various protozoa, such as *Leishmania amazonensis* and *Plasmodium falciparum* [17,18,19]. 

**Figure 5:**
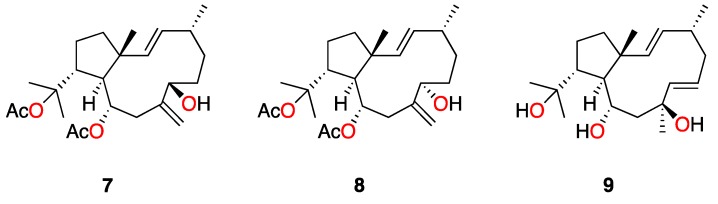
Structure of active dolabellanes isolated from the genus *Dictyota*.

In this study, the new dolabellane aldehyde, spiralyde A (**1**), showed the best IC_50_ values of antiprotozoal activity against *Trypanosoma cruzi* and *Leishmania amazonensis*. Compound **2** also showed moderate activity against both parasites. However, **3** and **4**, which only differ from **2** in the oxygenated substitution at C-14, were inactive. The analysis of the structure of all tested metabolites seems to indicate that the absence of an oxygenated function in the five-member ring of dolabellanes is relevant to obtain antikinetoplastid activity, as is the case of **1** and **2**, which lack a substituent at C-14 compared to **3**–**6**. This conclusion agrees with the absence of antibacterial activity reported for a family of dolabellanes with a ketone functionality at C-14 [9]. Other examples are the anti-VIH-1 epimers, dolabelladienols A (**7**) and B (**8**) [20], or the antileishmanicidal dolabelladienetriol (**9**) (IC_50_ of 44 µM on promastigotes) [19], molecules that lack substituents at C-14 of the dolabellane skeleton (Figure 5). Additionally, the presence of an α,β-unsaturated aldehyde functionality in spiralyde A (**1**), which may act as a Michael acceptor, seems to result in an enhanced antikinetoplastid activity with respect to **2**. The existence of Michael acceptor moieties both in natural products and synthetic compounds is considered a key feature due to the biological effects that these compounds usually display [21,22].

## 4. Materials and Methods 

### 4.1. General Experimental Procedures

Optical rotations were measured in CH>_2_Cl_2_ on a PerkinElmer 241 polarimeter (Waltham, MA, USA) by using a Na lamp. NMR spectra were recorded on a Bruker AVANCE 500 MHz or 600 MHz (Bruker Biospin, Falländen, Switzerland), as required. NMR spectra were obtained dissolving samples in CDCl3 (99.9%) and chemical shifts are reported relative to solvent (δH 7.26 and δC 77.0 ppm). Bruker AVANCE 600 MHz instrument is equipped with a 5 mm TCI inverse detection cryoprobe (Bruker Biospin, Falländen, Switzerland). Standard Bruker NMR pulse sequences were utilized. HR-ESI-MS data were obtained on an Waters LCT Premier XE Micromass (Manchester, UK) and VG -AutoSpec Micromass spectrometers (Manchester, UK), respectively. IR spectra were recorded on a Bruker IFS66/S (Ettlingen, Germany) equipped with an ATR accessory using CH_2_Cl_2_ solutions. EnSpire^®^ Multimode Reader (Perkin Elmer, Waltham, MA, USA) using absorbance values of Alamar Blue^®^ reagent (Bio-Rad Laboratories, Oxford, UK). HPLC (High performance liquid chromatography) separations were carried out with an Agilent 1260 Infinity Quaternary LC equipped with a Diode Array Detector (Waldbronn, Germany). TLC (Thin layer chromatography) (Merck, Darmstadt, Germany) was visualized by UV light (254 nm) and spraying with cobalt chloride reagent (2% in sulfuric acid, 10%) and heating.

### 4.2. Biological Material 

*Dictyota spiralis* [23] was collected in April 2017 off the coast of Tabarka, Northwest of Tunisia (36°57′37.6′′ N 8°45′13.2′′ E), at a depth not exceeding 1.5 m. The seaweed was harvested and transported in a cool box to the laboratory where it was cleaned, rinsed and dried at 40 °C in the dark. Dry material was powdered and used for extraction. Identification was made in the Laboratory of Blue Biotechnology and Aquatic Bioproducts (INSTM, Salammbô, Tunisia) and voucher specimens are kept at the laboratory of the National Institute of Marine Sciences and Technologies (INSTM, Tunisia) under the codes OC-04042017-1, OC-13042017-1, OC-17042017-1.

### 4.3. Extraction and Isolation

The dried and powdered algal material (190 g) was extracted by maceration at room temperature in dichloromethane (DCM). The solvent was renewed several times for a maximized extraction. DCM solution was filtered and evaporated with rotatory evaporator at 40 °C to give 10 g of crude extract. 5 g of the obtained extract was fractionated in a Sephadex LH-20 column, eluting with *n*-hexane, DCM and methanol (3:1:1) to give 7 fractions: F1 (0.86 g), F2 (0.743 g), F3 (1.131 g), F4 (1.775 g), F5 (0.283 g), F6 (0.195 g), F7 (0.062 g). Thin layer chromatography (TLC) monitoring was used with cobalt chloride (2%) as spraying reagent.

Fraction F3 (1.131 g) was further fractionated on a silica column, eluting with increasing polarity mixtures of *n*-hexane/ethyl acetate (EtOAc) (from 9:1 to 1:1, then 100% EtOAc) to furnish 10 subfractions, F3-1 to F3-10. Subfraction F3-1 (328.8 mg) was applied on a Lobar LiChroprep Si 60 (40–63 µm) column using a step-gradient *n*-hexane/EtOAc from 97:3 to 85:15, to obtain pure compounds: **6** (3.38 mg); **5** (111.64 mg) and **2** (36.11 mg). Subfraction F3-3 (355.55 mg) was chromatographed under the same conditions to isolate compound **4** (181.3 mg).

Fraction F5 (283 mg) was fractionated through an open silica gel column, using a gradient of *n*-hexane/EtOAc (8:2 to 1:1) and finally 100% EtOAc. A TLC monitoring allowed us to obtain 15 subfractions (F5-1 to F5-15); among them, F5-9 contained **3** (53.20 mg). HPLC purification of fraction F5-4 (9.04 mg) (Luna 5µm Silica (2) column, 100 Å, 250 × 10 mm, *n*-hexane/EtOAc, isocratic 9:1 for 10 min, gradient to 7:3 in 30 min, 7:3 for 30 min) led to the isolation of spiralyde A (**1**) (1.03 mg) (Supplementary Materials, Scheme S1).

#### 4.3.1. Spiralyde A (**1**)

Colorless oil; ${\left[\alpha \right]}_{D}^{20}$ +21 (*c* 0.05, CH_2_Cl_2_); UV (CH_2_Cl_2_) λ_max_ (log ε) 258 (2.69) nm; IR υ_max_ 3243, 2954, 2362, 2341, 1967, 1469, 1213 cm^−1^; HRESIMS *m***/***z* 325.2149 [M + Na]^+^ (calc. for 325.2144 C_20_H_30_O_2_Na); ^1^H and ^13^C NMR data (Table 2).

#### 4.3.2. (1*R*,3*S,*4*S*,7*E*,11*S*,12*S*)-3,4-Epoxy-7,18-dolabelladiene (**2**) 

Colorless oil; ${\left[\alpha \right]}_{D}^{20}$ +60 (*c* 3.44, CH_2_Cl_2_); HRESIMS *m*/*z* 311.2349 [M + Na]^+^ (calc. for 311.2351, C_20_H_32_ONa); ^1^H and ^13^C NMR spectra [9], see Supplementary Materials (Figures S9 and S10).

#### 4.3.3. (1*R*,3*S,*4*S*,7*E*,11*S*,12*S*,14*S*)-3,4-Epoxy-14-hydroxy-7,18-dolabelladiene (**3**) 

Yellow oil; ${\left[\alpha \right]}_{D}^{20}$ +51 (*c* 0.63 CH_2_Cl_2_); HRESIMS *m*/*z* 327.2294 [M + Na]^+^ (calc. for 327.2300, C_20_H_32_O_2_Na); ^1^H [10,11] and ^13^C NMR spectra [9,11], see Supplementary Materials (Figures S11 and S12).

#### 4.3.4. (1*R*,3*S,*4*S*,7*E*,11*S*,12*S*)-3,4-Epoxy-14-oxo-7,18-dolabelladiene (**4**) 

White amorphous solid; ${\left[\alpha \right]}_{D}^{20}$ +72 (*c* 3.16, CH_2_Cl_2_); HRESIMS *m*/*z* 325.2146 [M + Na]^+^ (calc. for 325.2144, C_20_H_30_O_2_Na); ^1^H and ^13^C NMR spectra [9], see Supplementary Materials (Figures S13 and S14).

#### 4.3.5. (1*R*,3*E*,7*E*,11*S*,12*S*)-14-Oxo-3,7,18-dolabellatriene (**5**) 

Colorless oil; ${\left[\alpha \right]}_{D}^{20}$ −40 (*c* 2.57, CH_2_Cl_2_); HRESIMS *m*/*z* 309.2196 [M + Na]^+^ (calc. for 309.2194, C_20_H_30_ONa); ^1^H and ^13^C NMR spectra [9], see Supplementary Materials (Figures S15 and S16).

#### 4.3.6. (1*R*,3*Z*,7*E*,11*S*,12*S*)-14-Oxo-3,7,18-dolabellatriene (**6**) 

Colorless oil; ${\left[\alpha \right]}_{D}^{20}$ –50 (*c* 0.40, CH_2_Cl_2_); HRESIMS *m*/*z* 309.2202 [M + Na]^+^ (calc. for 309.2194, C_20_H_30_ONa); ^1^H and ^13^C NMR spectra [9], see Supplementary Materials (Figures S17 and S18).

### 4.4. Evaluation of Leishmanicidal, Trypanocidal and Cytotoxic Activities

#### 4.4.1. Parasite Strains 

The activity of the crude extract, different subfractions and isolated compounds were evaluated against promastigotes of *Leishmania amazonensis* (MHOM/BR/77/LTB0016) and epimastigotes *Trypanosoma cruzi* (Y strain). Cytotoxicity assay of the active compounds was tested against the murine macrophage J774.A1 cell line (ATCC # TIB-67). 

#### 4.4.2. Leishmanicidal Capacity Assay

Logarithmic phase cultures of *Leishmania amazonensis* were used for experimental purposes, and the in vitro susceptibility assay was performed in sterilized 96-well plates. 10^6^/well parasites were added to wells containing different concentration of the drug to be tested. Percentages of inhibition, 50% inhibitory concentrations (IC_50_) for active compounds were calculated by linear regression analysis using the Alamar Blue method [24]. 

#### 4.4.3. Trypanocidal Capacity Assay 

The activity was evaluated in vitro against epimastigote stage of *Trypanosoma cruzi.* Different concentrations of fractions and compounds were incubated in 96 wells plate for 96 h with a density of 10^5^ parasite/well. 10% of Alamar blue was added to each well and the IC_50_ was calculated. All assays have been realized in triplicate.

#### 4.4.4. Cytotoxicity Assay 

The cytotoxicity of active compounds was evaluated in murine macrophage J774.A1 cell line (ATCC # TIB-67). Different concentrations were incubated for 24 h and viability was determined with the Alamar Blue method using dose-response curves to obtain the CC_50_ [25].

## 5. Conclusions

Considered as neglected tropical diseases, leishmaniasis and Chagas disease affects millions of people worldwide, however, first line existing treatments are not satisfactory mainly due to drug resistance, lack of effectiveness and toxicity [3,5].

**Figure 6:**
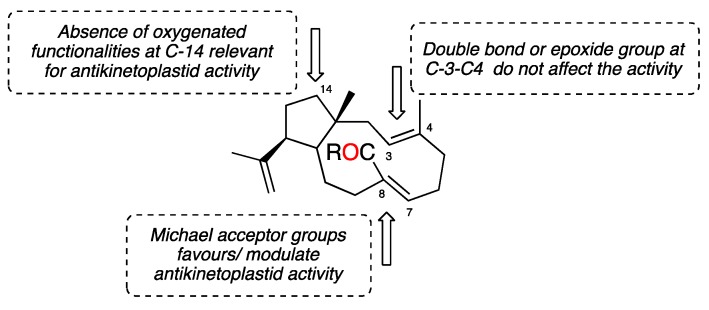
Preliminar structure-activity relationship on antikinetoplastid activity of *Dictyota* dolabellanes.

To the best of our knowledge, there is only one previously reported dolabellane from *Dictyota* species that possesses moderate antiprotozoal activity against the promastigote form of *L. amazonensis*, dolabelladientriol (**9**), (IC_50_ 44 mM) [19], whereas none has been reported against *Trypanosoma*. In this bioassay-guided study we have identified two active dolabellane diterpenes, **1**–**2**, against the kinetoplastids *Trypanosoma cruzi* and *Leishmania amazonensis*, together with the inactive dolabellanes **3**–**6**. The new compound spiralyde A (**1**) has showed to be the most active (IC_50_ 5.62 μM, against the epimastigote form of *T. cruzi*), comparable to benznidazole, the current commercial drug against *Trypanosoma*, and good activity against *Leishmania* (IC_50_ 15.47 μM). As summarized in Figure 6, a preliminary SAR analysis of metabolites **1**–**6** seems to indicate that the absence of substituents in the five-member ring of dolabellanes is relevant to obtain antikinetoplastid activity, in particular oxygenated functionalities at C-14. On the other hand, the enhanced activity of spiralyde A (**1**) with respect to the active **2** points out the fact that the presence of a Michael acceptor fragment located at C-7-C-8 double bond may be key to modulating the biological effect. Our results suggest dolabellane diterpenes as candidates to be a potential source of novel therapeutic agents against antikinetoplastid parasites. 

## Figures and Tables

**Table 1:** Antikinetoplastid activity of the organic extract and active fractions of *Dictyota spiralis*.

Sample	*Leishmania amazonensis* IC_50_ (µg/mL)	*Trypanosoma cruzi* IC_50_ (µg/mL)
Crude extract	9.76 ± 0.55	8.82 ± 0.98
F3	7.54 ± 0.84	15.52 ± 1.99
F5	15.5 ± 0.83	5.78 ± 1.71

**Table 2:** NMR spectroscopic data for spiralyde A (**1**) (CDCl_3_, 300 K, 600 MHz).

Position	Spiralyde A (1)	
	δ_C_, Type	δ_H_ (*J* in Hz)
1	47.1, C	
2	42.3, CH_2_	2.20, m 1.68, m
3	127.4, CH	5.33, dd (11.7, 5.2)
4	134.1 ^a^, C	
5	39.2, CH_2_	α: 2.38, m β: 2.30, dddd (14.7, 8.7, 4.1, 2.7)
6	23.7, CH_2_	α: 3.03, m β: 2.42, m
7	153.9, CH	6.31 dd (12.6, 3.7)
8	139.3 ^a^, C	
9	76.9 ^a^, CH	4.30 ddd (13.0, 6.0, 3.0)
10	33.5, CH_2_	α: 1.50, m β: 1.69, m
11	42.1, CH	1.42, m
12	51.4, CH	2.49 ddd (12.6, 6.7, 6.7)
13	27.7, CH_2_	1.60, m 1.52, m
14	41.5, CH_2_	α: 1.45, m β: 1.53, m
15	24.9, CH_3_	1.11, s
16	15.8, CH_3_	1.55, s
17	191.8, CH	10.02 d (2.0)
18	145.6 ^a^, C	
19	23.1, CH_3_	1.53, s
20	111.4, CH_2_	4.87, s 4.65, s

^a^ Chemical shift deduced from the HSQC and/or HMBC experiments.

**Table 3:** Antikinetoplastic effect of dolabellanes **1**–**6** against *Leishmania amazonensis* and *Trypanosoma cruzi (*IC_50_). Toxicity against murine macrophage J774.A1 *(*CC_50_). * Reference compounds.

Sample	*Leishmania amazonensis* IC_50_ (µM)	*Trypanosoma cruzi* IC_50_ (µM)	*Macrophage J774* CC_50_ (µM)
Spiralyde A (**1**)	15.47 ± 0.26	5.62 ± 2.48	23.4 ± 5.62
**2**	36.81 ± 5.20	35.29 ± 4.09	69.98± 0.14
**3**	>100	>100	-
**4**	>100	>100	-
**5**	>100	>100	-
**6**	>100	>100	-
Miltefosine *	6.48 ± 0.24	-	72.19 ± 3.06
Benznidazole *	-	6.94 ± 1.94	400 ± 4.00

## Supplementary Materials

The following are available online at https://www.mdpi.com/1660-3397/17/3/192/s1, Scheme S1, Bioassay-guided fractionation process of *Dictyota spiralis*, Figure S1: ^1^H NMR spectrum of spiralyde A (**1**) (600 MHz, CDCl_3_),Figure S2: ^13^C NMR spectrum of spiralyde A (**1**) (150 MHz, CDCl_3_), Figure S3: ^1^H-^1^H COSY spectrum of spiralyde A (**1**) (600 MHz, CDCl_3_), Figure S4: HSQC spectrum of spiralyde A (**1**) (600 MHz, CDCl_3_), Figure S5: HMBC spectrum of spiralyde A (**1**) (600 MHz, CDCl_3_), Figure S6: ROESY spectrum of spiralyde A (**1**) (600 MHz, CDCl_3_), Figure S7: H-9 long-range COSY couplings of spiralyde A (**1**) (600 MHz, CDCl_3_), Figure S8: LRESIMS spectrum of spiralyde A (**1**), Figure S9: HRESIMS spectrum of spiralyde A (**1**), Figure S10: ^1^H NMR spectrum of (1*R*,3*S,*4*S*,7*E*,11*S*,12*S*)-3,4-Epoxy-7,18-dolabelladiene (**2**) (500 MHz, CDCl_3_), Figure S11: ^13^C NMR spectrum of (1*R*,3*S,*4*S*,7*E*,11*S*,12*S*)-3,4-Epoxy-7,18-dolabelladiene (**2**) (125 MHz, CDCl_3_), Figure S12: ^1^H NMR spectrum of (1*R*,3*S,*4*S*,7*E*,11*S*,12*S*,14*S*)-3,4-Epoxy-14-hydroxy-7,18-dolabelladiene (**3**) (500 MHz, CDCl_3_), Figure S13: ^13^C NMR spectrum of (1*R*,3*S,*4*S*,7*E*,11*S*,12*S*,14*S*)-3,4-Epoxy-14-hydroxy-7,18-dolabelladiene (**3**) (125 MHz, CDCl_3_), Figure S14: ^1^H NMR spectrum of (1*R*,3*S,*4*S*,7*E*,11*S*,12*S*)-3,4-Epoxy-14-oxo-7,18-dolabelladiene (**4**) (500 MHz, CDCl_3_), Figure S15: ^13^C NMR spectrum of (1*R*,3*S,*4*S*,7*E*,11*S*,12*S*)-3,4-Epoxy-14-oxo-7,18-dolabelladiene (**4**) (125 MHz, CDCl_3_), Figure S16: ^1^H NMR spectrum of (1*R*,3*E*,7*E*,11*S*,12*S*)-14-oxo-3,7,18-dolabellatriene (**5**) (500 MHz, CDCl_3_), Figure S17: ^13^C NMR spectrum of (1*R*,3*E*,7*E*,11*S*,12*S*)-14-oxo-3,7,18-dolabellatriene (**5**) (125 MHz, CDCl_3_), Figure S18: ^1^H NMR spectrum of (1*R*,3*Z*,7*E*,11*S*,12*S*)-14-oxo-3,7,18-dolabellatriene (**6**) (500 MHz, CDCl_3_), Figure S19: ^13^C NMR spectrum of (1*R*,3*Z*,7*E*,11*S*,12*S*)-14-oxo-3,7,18-dolabellatriene (**6**) (125 MHz, CDCl_3_).
