# Supplementary material for: Spiralyde A, an Antikinetoplastid Dolabellane from the Brown Alga Dictyota spiralis
Source: Mar Drugs. 2019 Mar 25;17(3):192. doi: 10.3390/md17030192 (PMC6471066; doi:10.3390/md17030192)

Supporting Information

Spiralyde A, an antikinetoplastid dolabellane from the brown algae *Dictyota spiralis*

Olfa Chiboub[1,2,3,4], Ines Sifaoui[2,3], Jacob Lorenzo-Morales[2], Manef Abderrabba[3], Mondher Mejri[3], José Javier Fernandez[1,5,*], José E. Piñero[2], and Ana R. Díaz-Marrero[1,*]

[1] Instituto Universitario de Bio-Orgánica Antonio González (IUBO AG), Centro de Investigaciones Biomédicas de Canarias (CIBICAN), Universidad de La Laguna (ULL), Avda. Astrofísico F. Sánchez, 2, 38206 La Laguna, Tenerife, España

[2] Instituto Universitario de Enfermedades Tropicales y Salud Pública de Islas Canarias, Universidad de La Laguna, Avda. Astrofísico F. Sánchez s/n, 38206 La Laguna, Tenerife, España

[3] Laboratoire Matériaux-Molécules et Applications, IPEST, B.P 51 2070, La Marsa, University of Carthage, Tunisia

[4] Institut National d'Agronomie de Tunis, 43 Avenue Charles Nicolle, Tunis 1082, University of Carthage, Tunisia

[5] Departamento de Química Orgánica, Universidad de La Laguna (ULL), Avda. Astrofísico F. Sánchez, 2, 38206 La Laguna, Tenerife, España

*Correspondence: jjfercas@ull.edu.es (J.J.F.); adiazmar@ull.edu.es (A.R.D.-M.); Tel.: +34-922-318-586 (J.J.F. & A.R.D.-M.)

TABLE OF CONTENTS

	Page
**Scheme S1.** Bioassay-guided fractionation process of *Dictyota spiralis*	S3
**Figure S1.** ^{1}H NMR spectrum of spiralyde A (**1**) (600 MHz, $CDCl_3$)	S4
**Figure S2.** ^{13}C NMR spectrum of spiralyde A (**1**) (150 MHz, $CDCl_3$)	S5
**Figure S3.** ^{1}H-^{1}H COSY spectrum of spiralyde A (**1**) (600 MHz, $CDCl_3$)	S6
**Figure S4.** HSQC spectrum of spiralyde A (**1**) (600 MHz, $CDCl_3$)	S7
**Figure S5.** HMBC spectrum of spiralyde A (**1**) (600 MHz, $CDCl_3$)	S8
**Figure S6.** ROESY spectrum of spiralyde A (**1**) (600 MHz, $CDCl_3$)	S9
**Figure S7.** H-9 long-range COSY couplings of spiralyde A (**1**) (600 MHz, $CDCl_3$)	S10
**Figure S8.** LRESIMS spectrum of spiralyde A (**1**)	S11
**Figure S9.** HRESIMS spectrum of spiralyde A (**1**)	S12
**Figure S10.** ^{1}H NMR spectrum of (1*R*,3*S*,4*S*,7*E*,11*S*,12*S*)-3,4-Epoxy-7,18-dolabelladiene (**2**) (500 MHz, $CDCl_3$)	S13
**Figure S11.** ^{13}C NMR spectrum of (1*R*,3*S*,4*S*,7*E*,11*S*,12*S*)-3,4-Epoxy-7,18-dolabelladiene (**2**) (125 MHz, $CDCl_3$)	S14
**Figure S12.** ^{1}H NMR spectrum of (1*R*,3*S*,4*S*,7*E*,11*S*,12*S*,14*S*)-3,4-Epoxy-14-hydroxy-7,18-dolabelladiene (**3**) (500 MHz, $CDCl_3$)	S15
**Figure S13.** ^{13}C NMR spectrum of (1*R*,3*S*,4*S*,7*E*,11*S*,12*S*,14*S*)-3,4-Epoxy-14-hydroxy-7,18-dolabelladiene (**3**) (125 MHz, $CDCl_3$)	S16
**Figure S14.** ^{1}H NMR spectrum of (1*R*,3*S*,4*S*,7*E*,11*S*,12*S*)-3,4-Epoxy-14-oxo-7,18-dolabelladiene (**4**) (500 MHz, $CDCl_3$)	S17
**Figure S15.** ^{13}C NMR spectrum of (1*R*,3*S*,4*S*,7*E*,11*S*,12*S*)-3,4-Epoxy-14-oxo-7,18-dolabelladiene (**4**) (125 MHz, $CDCl_3$)	S18
**Figure S16.** ^{1}H NMR spectrum of (1*R*,3*E*,7*E*,11*S*,12*S*)-14-oxo-3,7,18-dolabellatriene (**5**) (500 MHz, $CDCl_3$)	S19
**Figure S17.** ^{13}C NMR spectrum of (1*R*,3*E*,7*E*,11*S*,12*S*)-14-oxo-3,7,18-dolabellatriene (**5**) (125 MHz, $CDCl_3$)	S20
**Figure S18.** ^{1}H NMR spectrum of (1*R*,3*Z*,7*E*,11*S*,12*S*)-14-oxo-3,7,18-dolabellatriene (**6**) (500 MHz, $CDCl_3$)	S21
**Figure S19.** ^{13}C NMR spectrum of (1*R*,3*Z*,7*E*,11*S*,12*S*)-14-oxo-3,7,18-dolabellatriene (**6**) (125 MHz, $CDCl_3$)	S21

Scheme S1. Bioassay-guided fractionation process of *Dictyota spiralis*

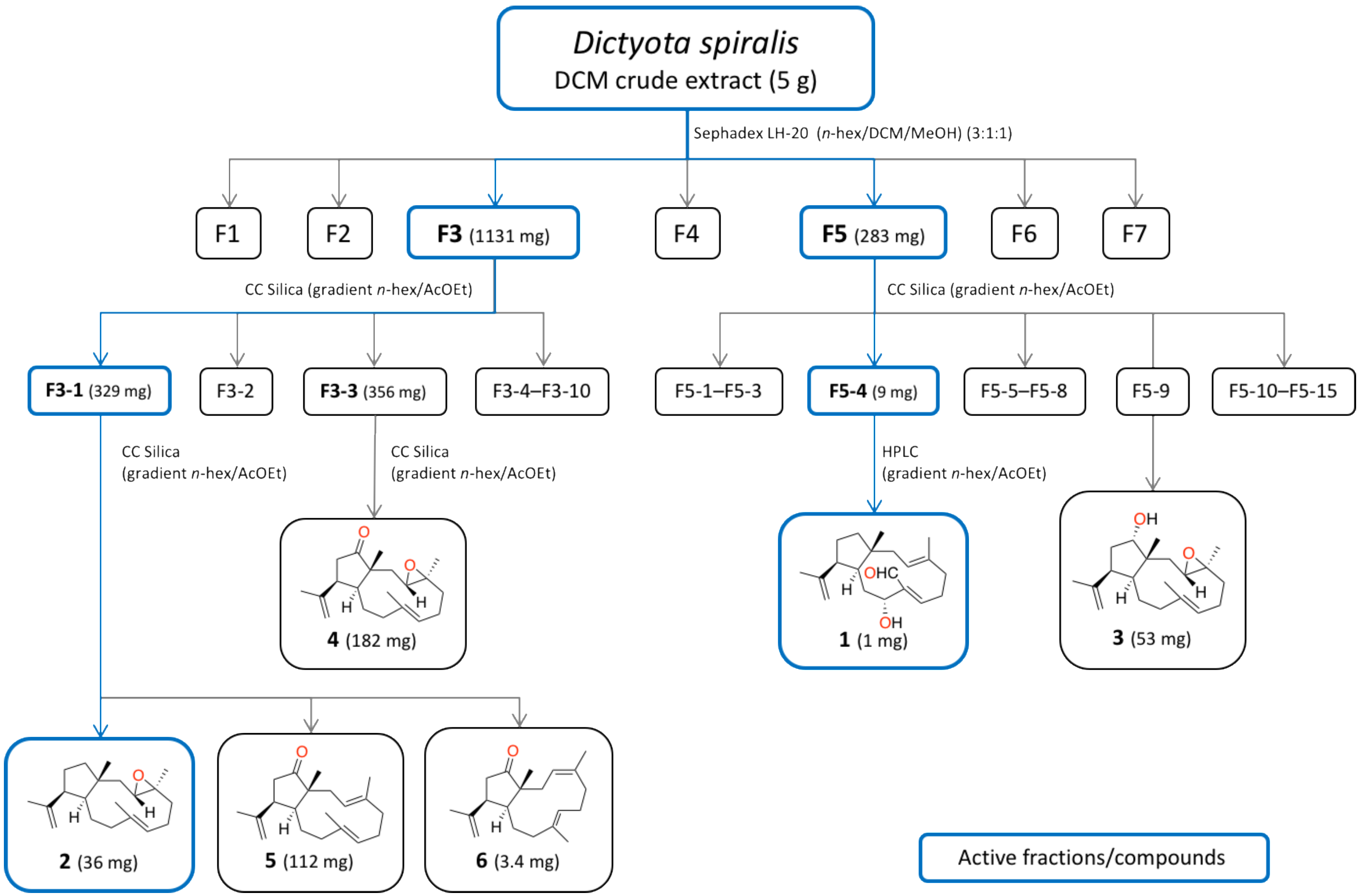

Figure S1. ¹H NMR spectrum of spiralyde A (**1**) (600 MHz, $CDCl_3$)

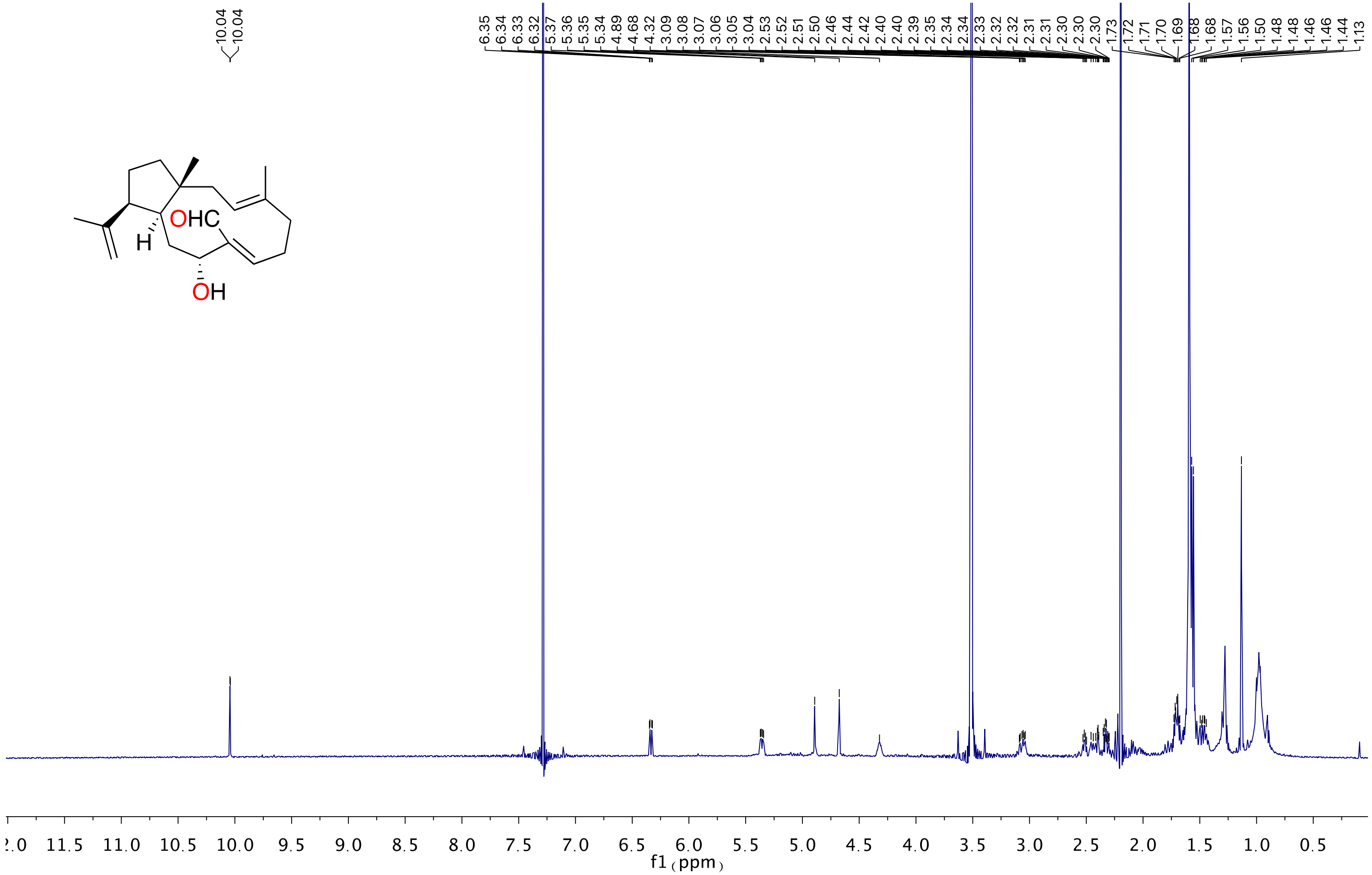

Figure S2. ^{13}C NMR spectrum of spiralyde A (**1**) (150 MHz, $CDCl_3$)

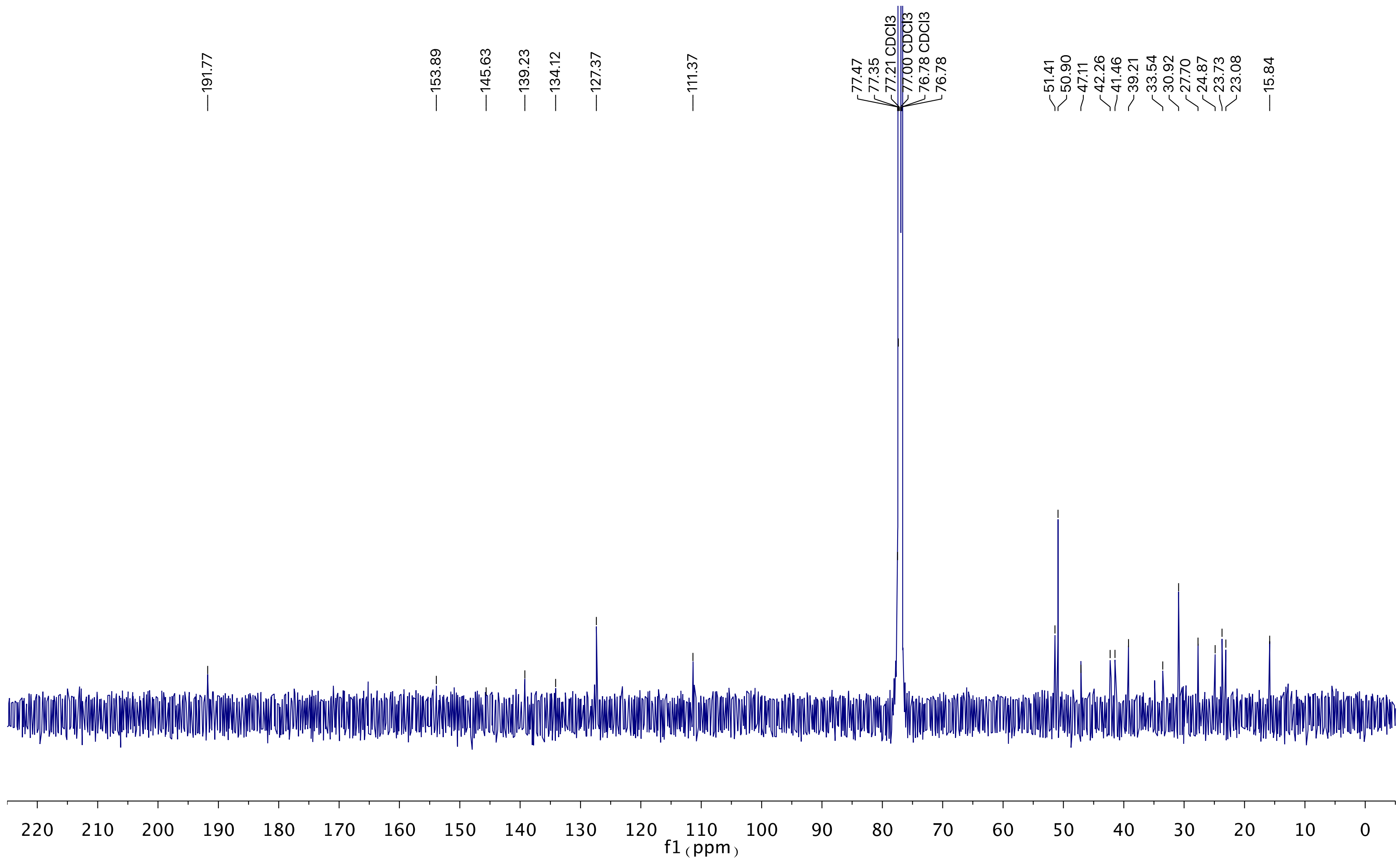

Figure S3. ^1H-^1H COSY spectrum of spiralyde A (**1**) (600 MHz, $CDCl_3$)

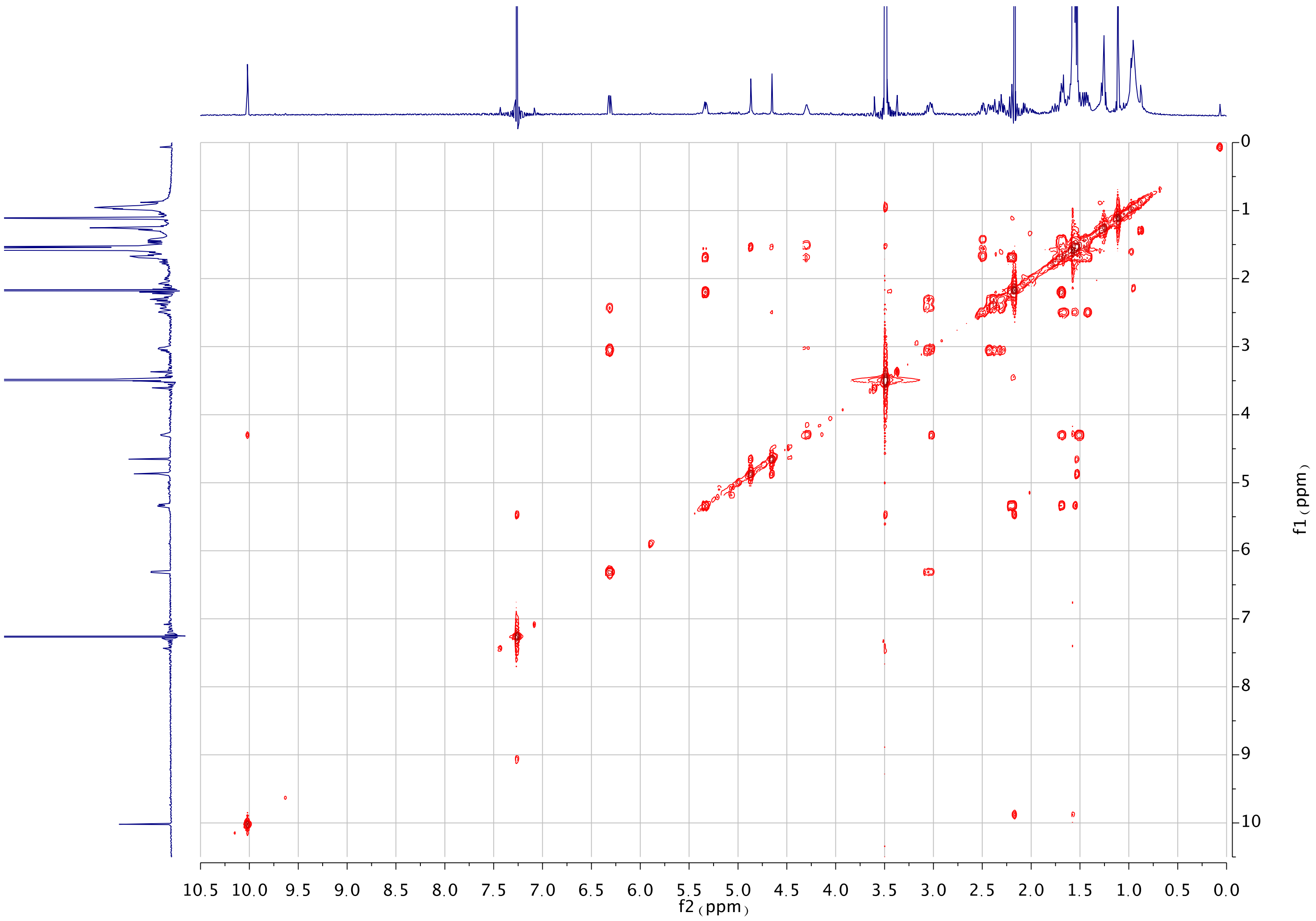

Figure S4. HSQC spectrum of spiralyde A (**1**) (600 MHz, $CDCl_3$)

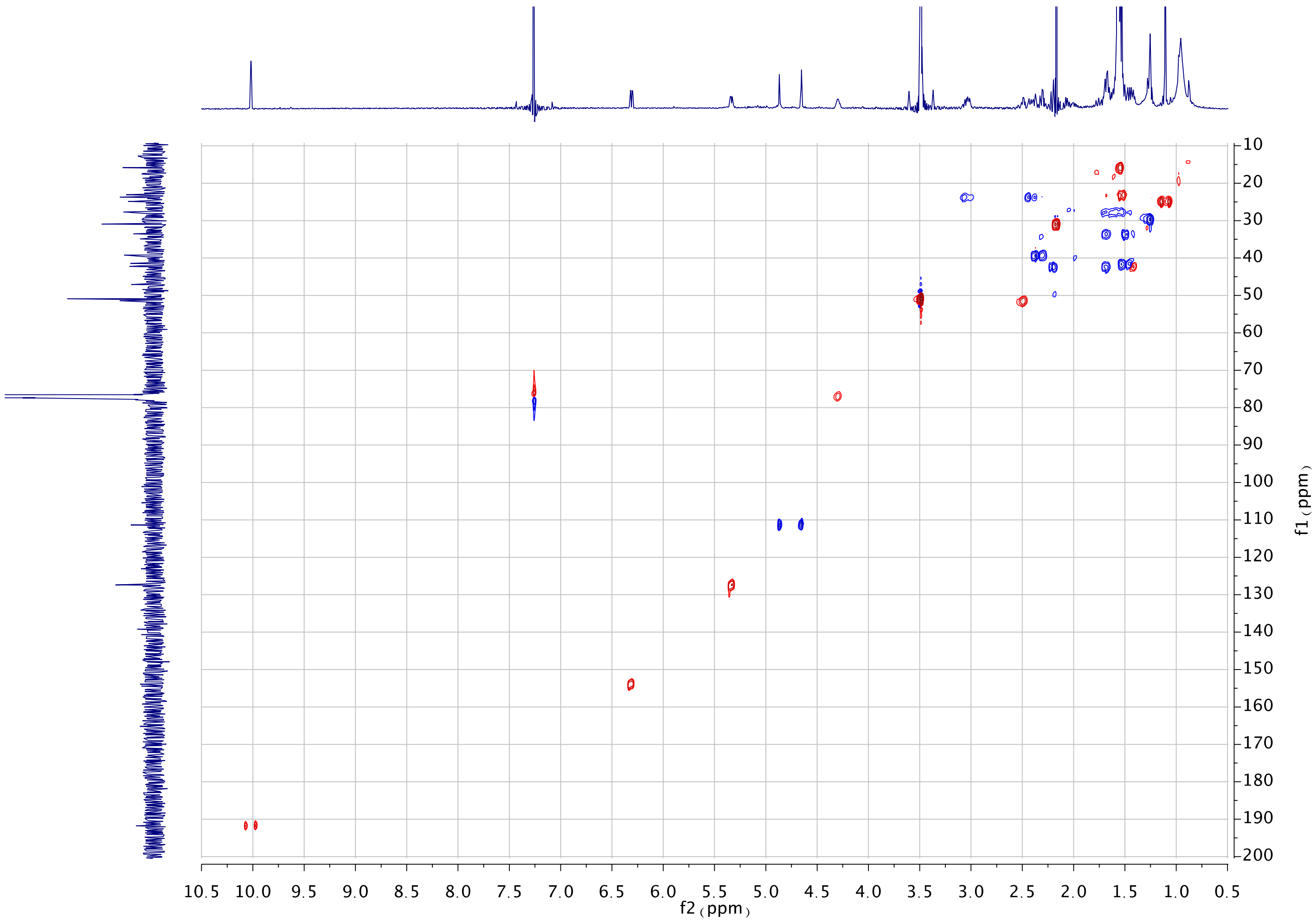

Figure S5. HMBC spectrum of spiralyde A (**1**) (600 MHz, $CDCl_3$)

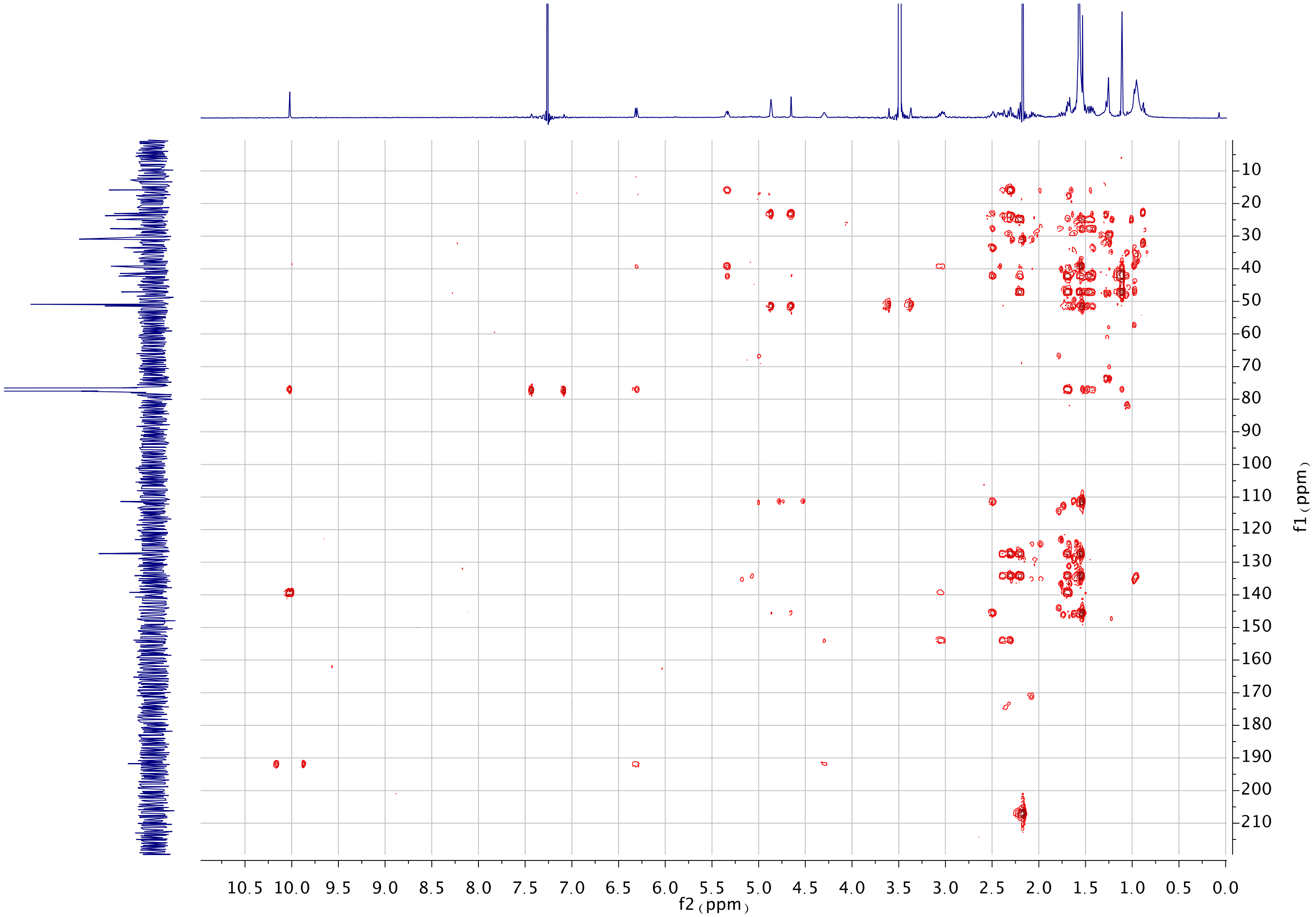

Figure S6. ROESY spectrum of spiralyde A (**1**) (600 MHz, $CDCl_3$)

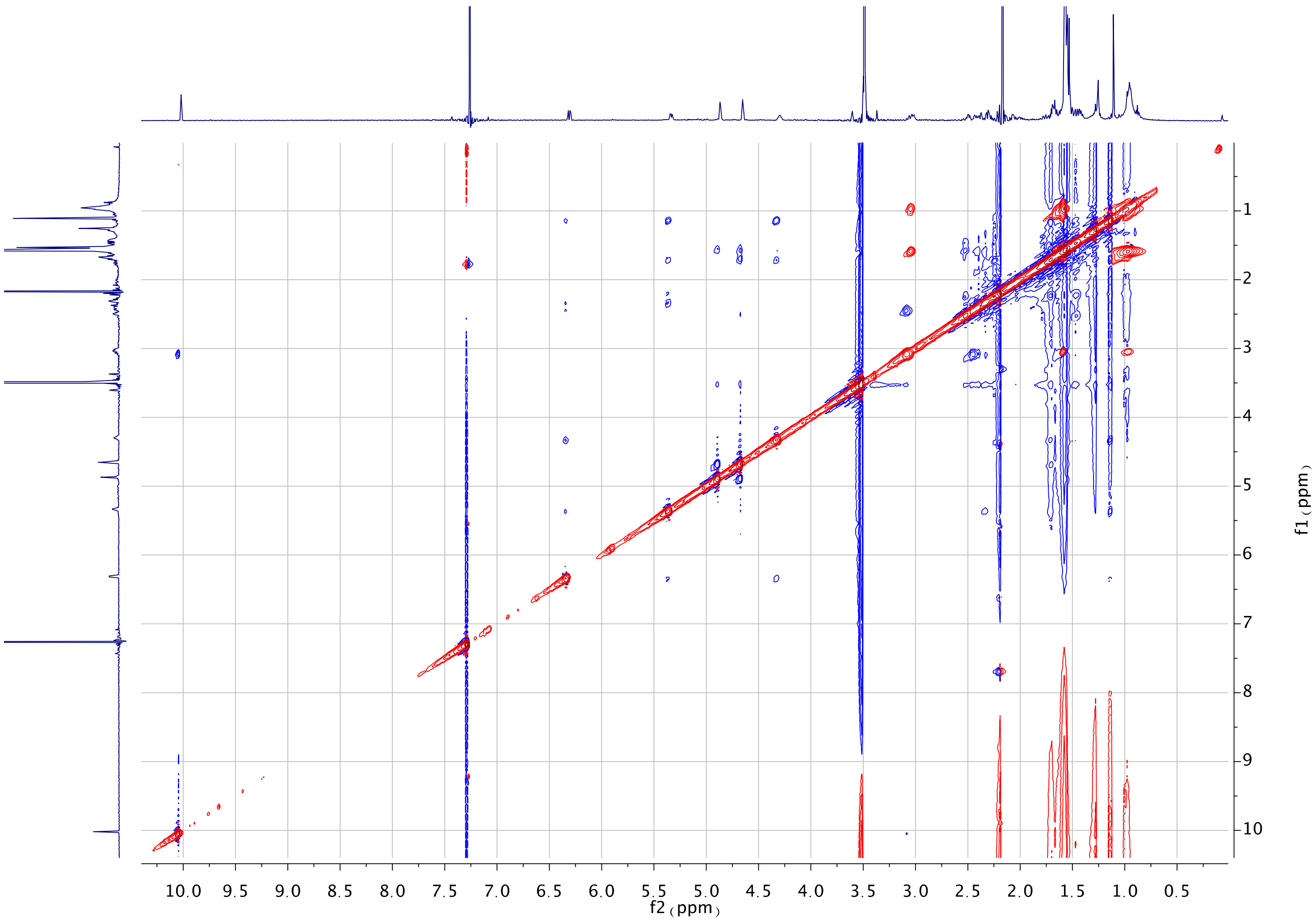

Figure S7. H-9 long-range COSY couplings of spiralyde A (**1**) (600 MHz, $CDCl_3$)

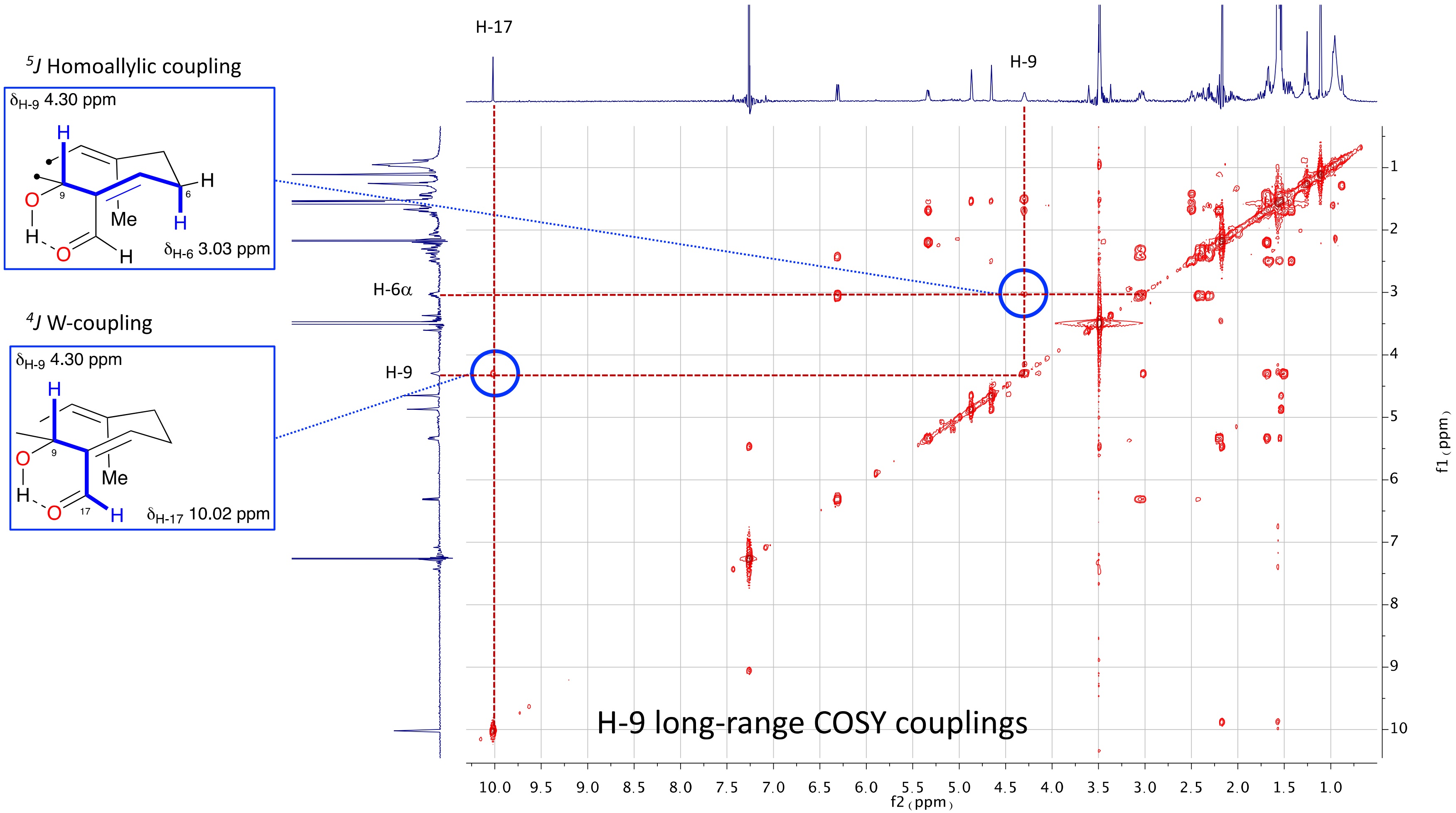

Figure S8. LRESIMS spectrum of spiralyde A (**1**)

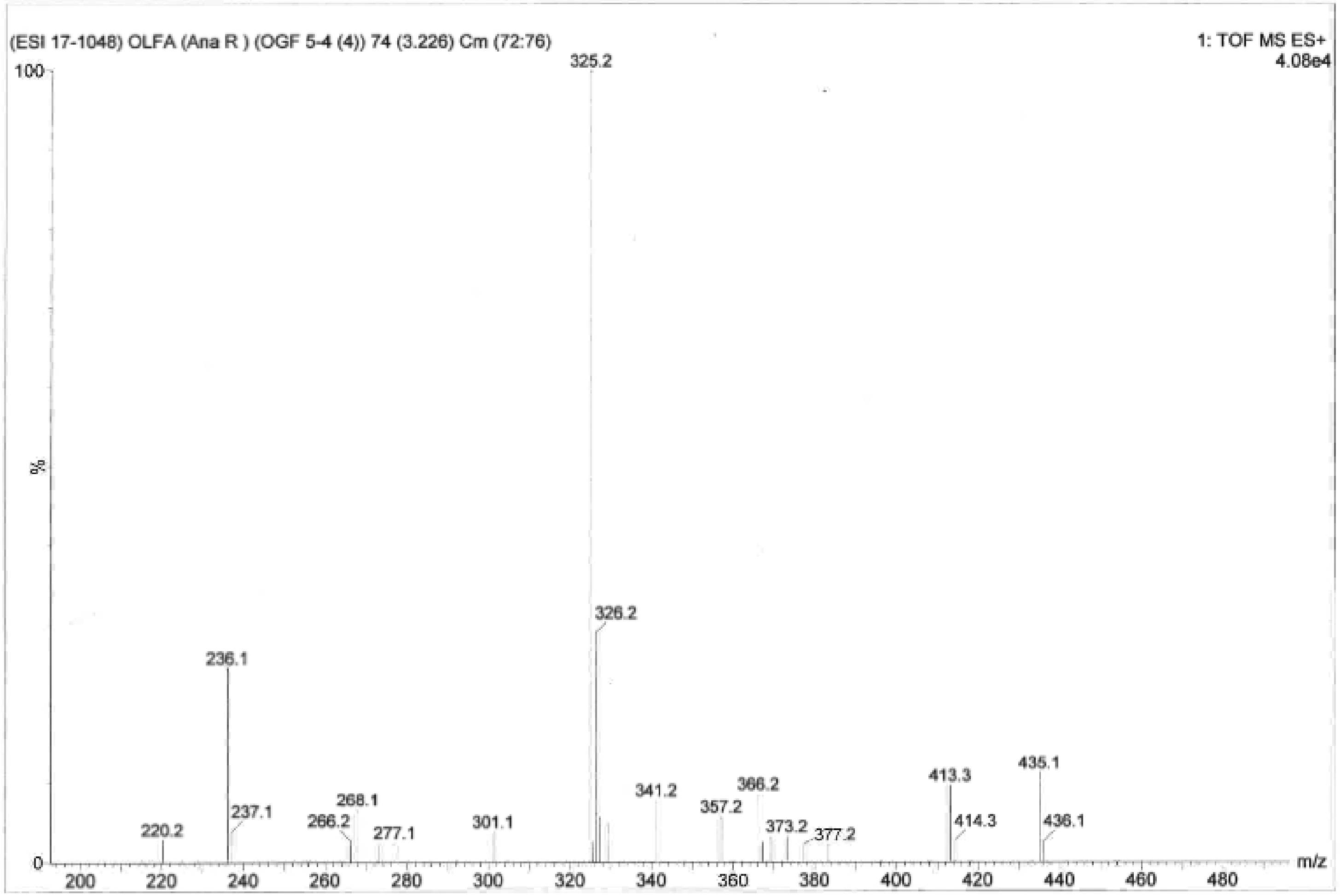

Figure S9. HRESIMS spectrum of spiralyde A (**1**)

Elemental Composition Report

Page 1

Tolerance = 5.0 PPM / DBE: min = -10.0, max = 1000.0
Element prediction: Off
Number of isotope peaks used for i-FIT = 2

Monoisotopic Mass, Even Electron Ions
1565 formula(e) evaluated with 1 results within limits (all results (up to 1000) for each mass)
Elements Used:
C: 0-128 H: 0-160 N: 0-1 O: 0-24 Na: 0-1 S: 0-4 133Cs: 0-1

(ESI 17-1048) OLFA (Ana R) (OGF 5-4 (4)) 40 (1.393)

2: TOF MS ES+
4.06e+003

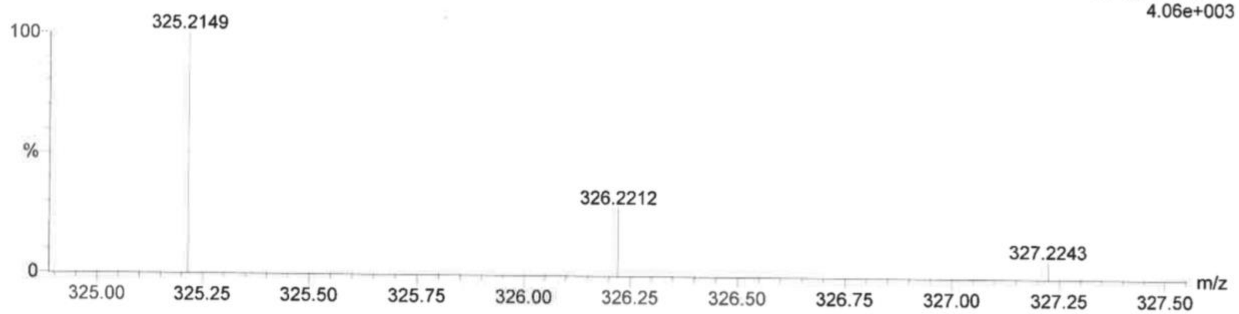

	Mass	RA	Calc. Mass	mDa	PPM	DBE	i-FIT	Formula
Minimum:		30.00				-10.0		
Maximum:		100.00		100.0	5.0	1000.0		
	325.2149	100.00	325.2144	0.5	1.5	5.5	17.9	C20 H30 O2 Na

Figure S10. ^{1}H NMR spectrum of (1*R*,3*S*,4*S*,7*E*,11*S*,12*S*)-3,4-Epoxy-7,18-dolabelladiene (**2**) (500 MHz, $CDCl_3$)

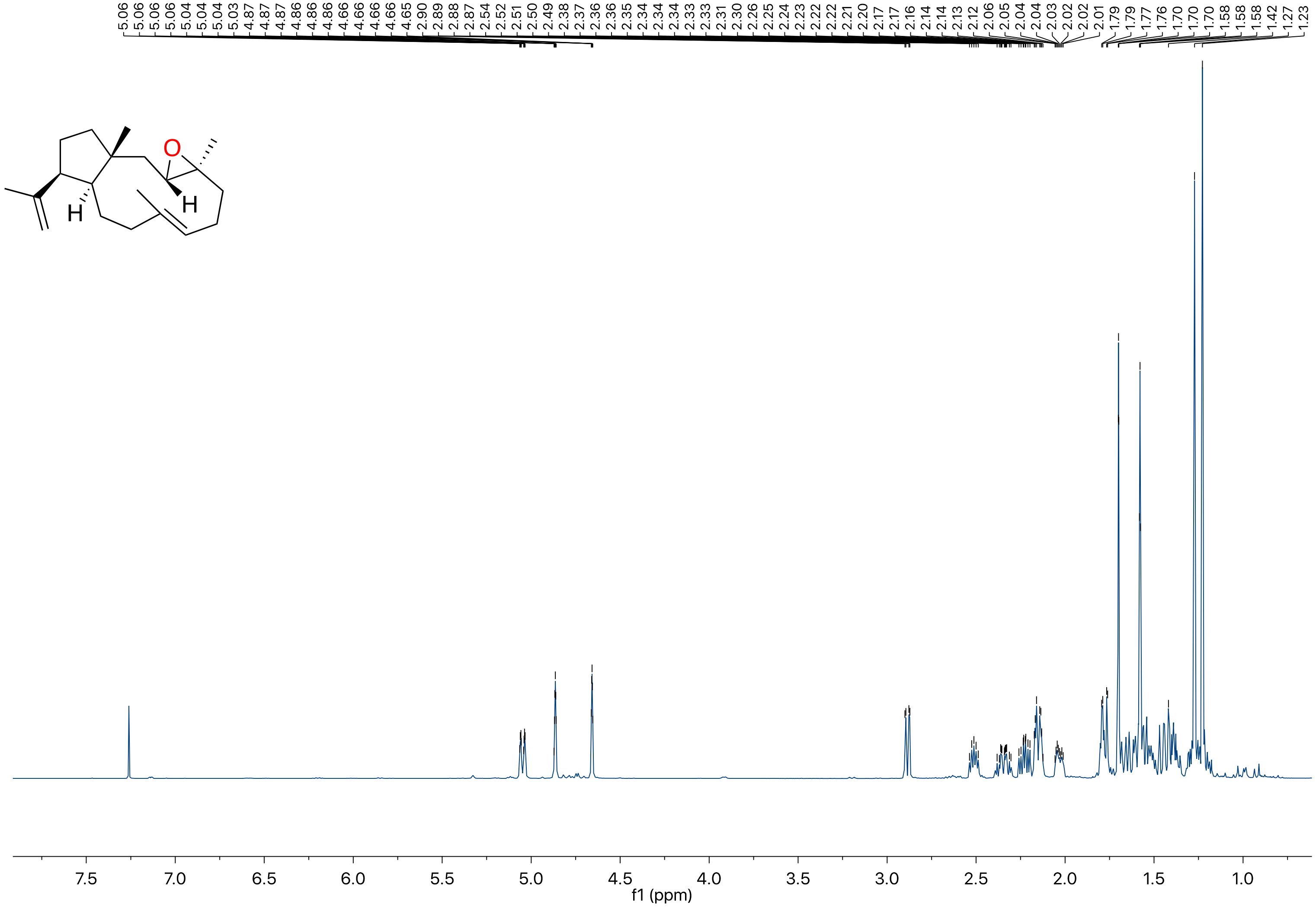

Figure S11. ^{13}C NMR spectrum of (1*R*,3*S*,4*S*,7*E*,11*S*,12*S*)-3,4-Epoxy-7,18-dolabelladiene (**2**) (125 MHz, $CDCl_3$)

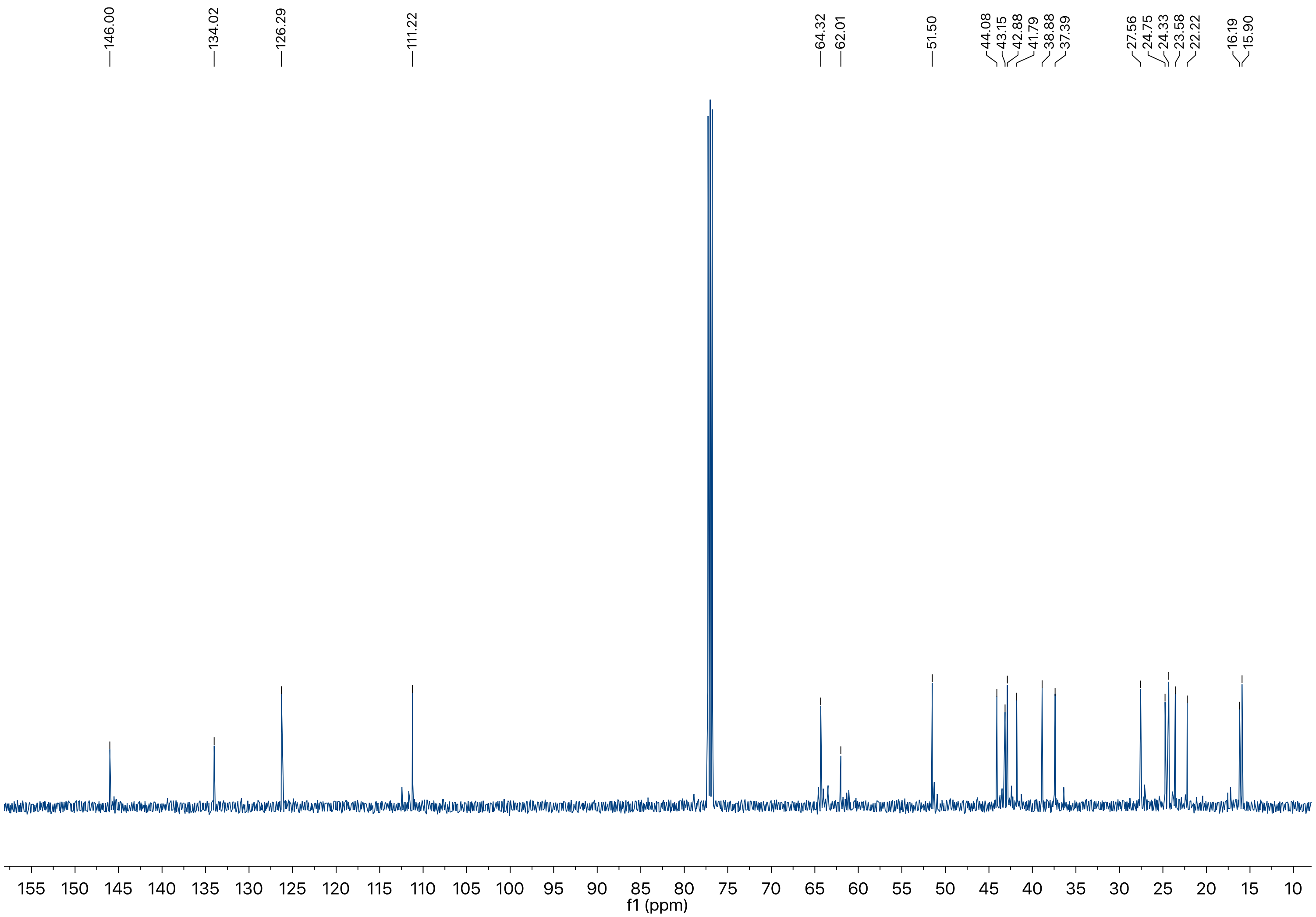

Figure S12. ^{1}H NMR spectrum of (1*R*,3*S*,4*S*,7*E*,11*S*,12*S*,14*S*)-3,4-Epoxy-14-hydroxy-7,18-dolabelladiene (**3**) (500 MHz, $CDCl_3$)

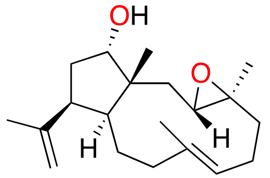

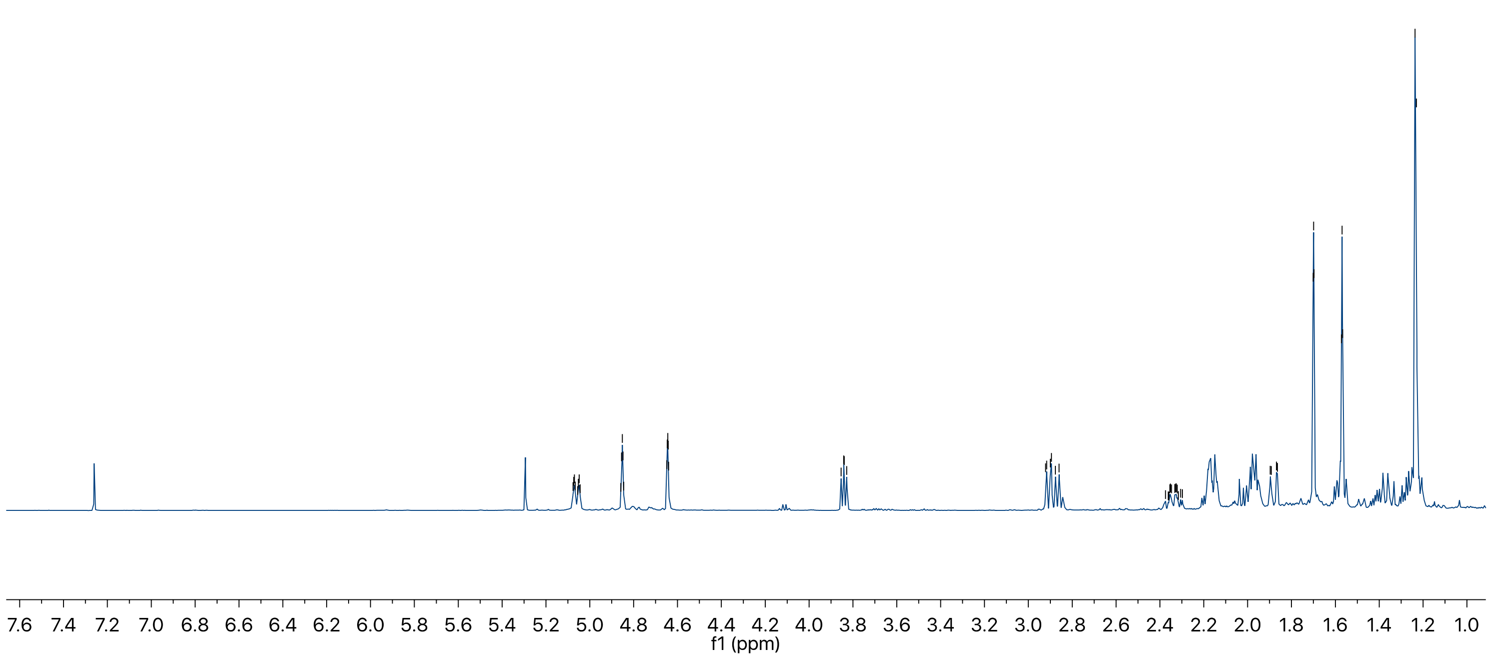

Figure S13. ^{13}C NMR spectrum of (1*R*,3*S*,4*S*,7*E*,11*S*,12*S*,14*S*)-3,4-Epoxy-14-hydroxy-7,18-dolabelladiene (**3**) (125 MHz, $CDCl_3$)

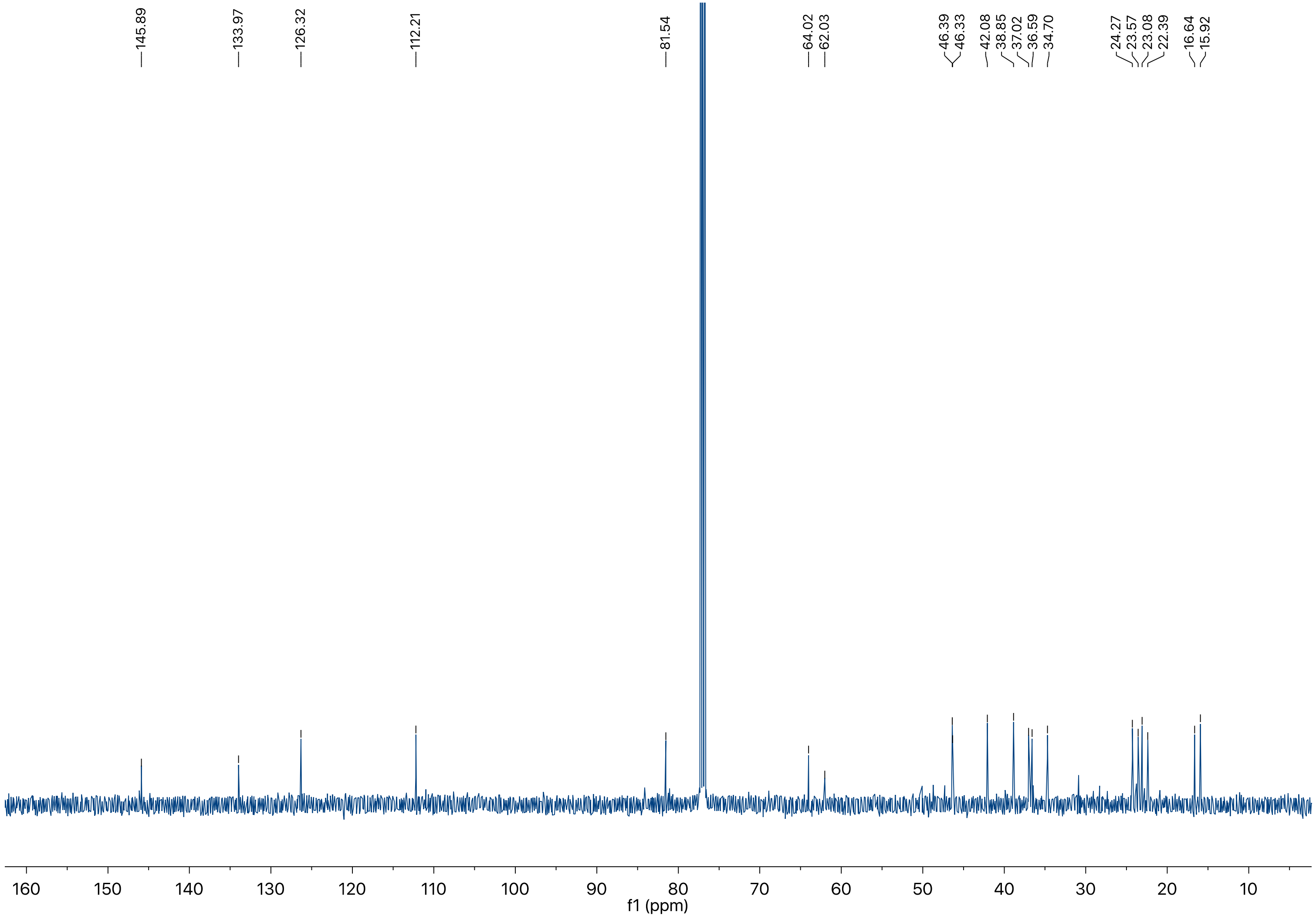

Figure S14. ^{1}H NMR spectrum of (1*R*,3*S*,4*S*,7*E*,11*S*,12*S*)-3,4-Epoxy-14-oxo-7,18-dolabelladiene (**4**) (500 MHz, $CDCl_3$)

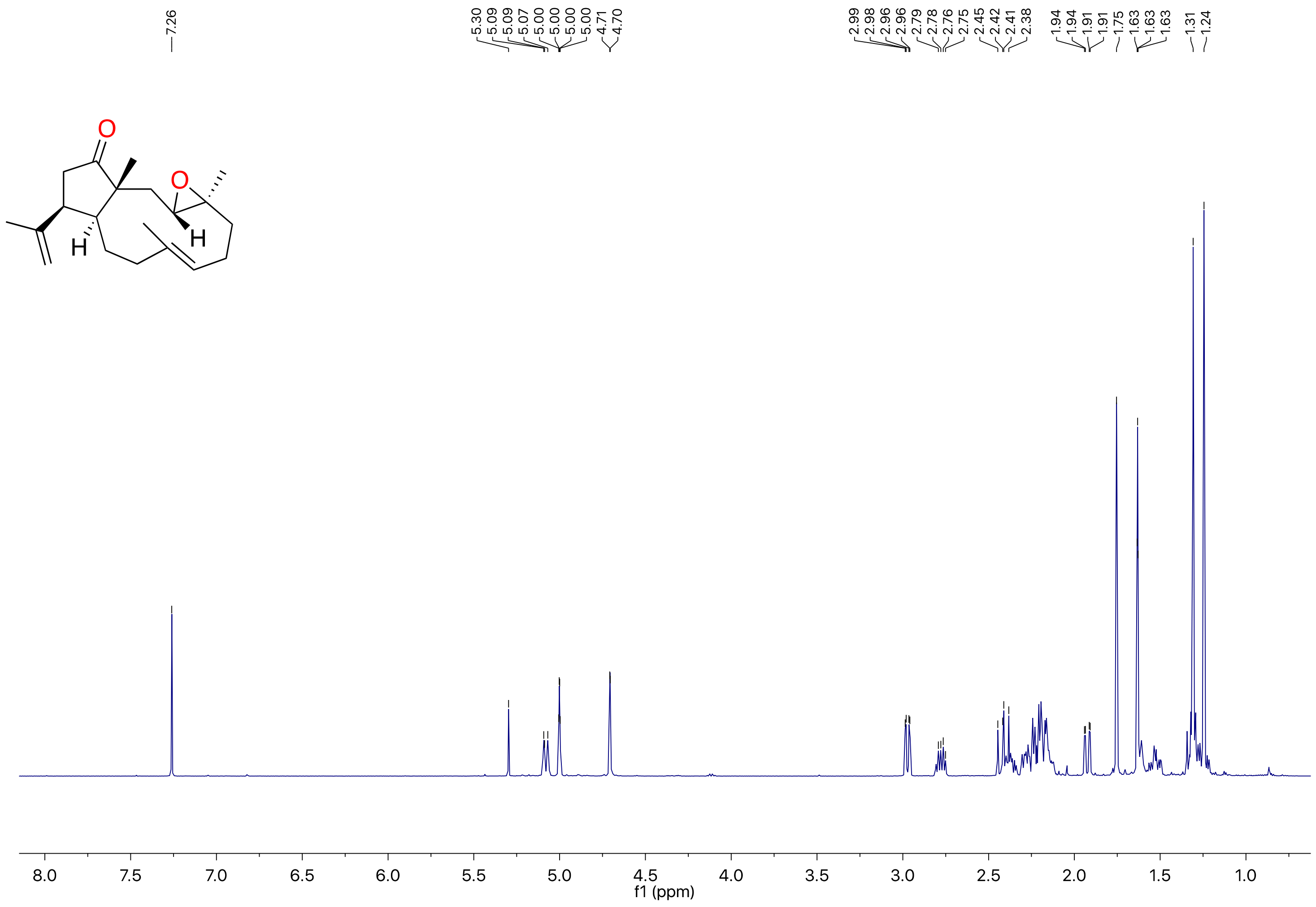

Figure S15. ^{13}C NMR spectrum of (1*R*,3*S*,4*S*,7*E*,11*S*,12*S*)-3,4-Epoxy-14-oxo-7,18-dolabelladiene (**4**) (125 MHz, $CDCl_3$)

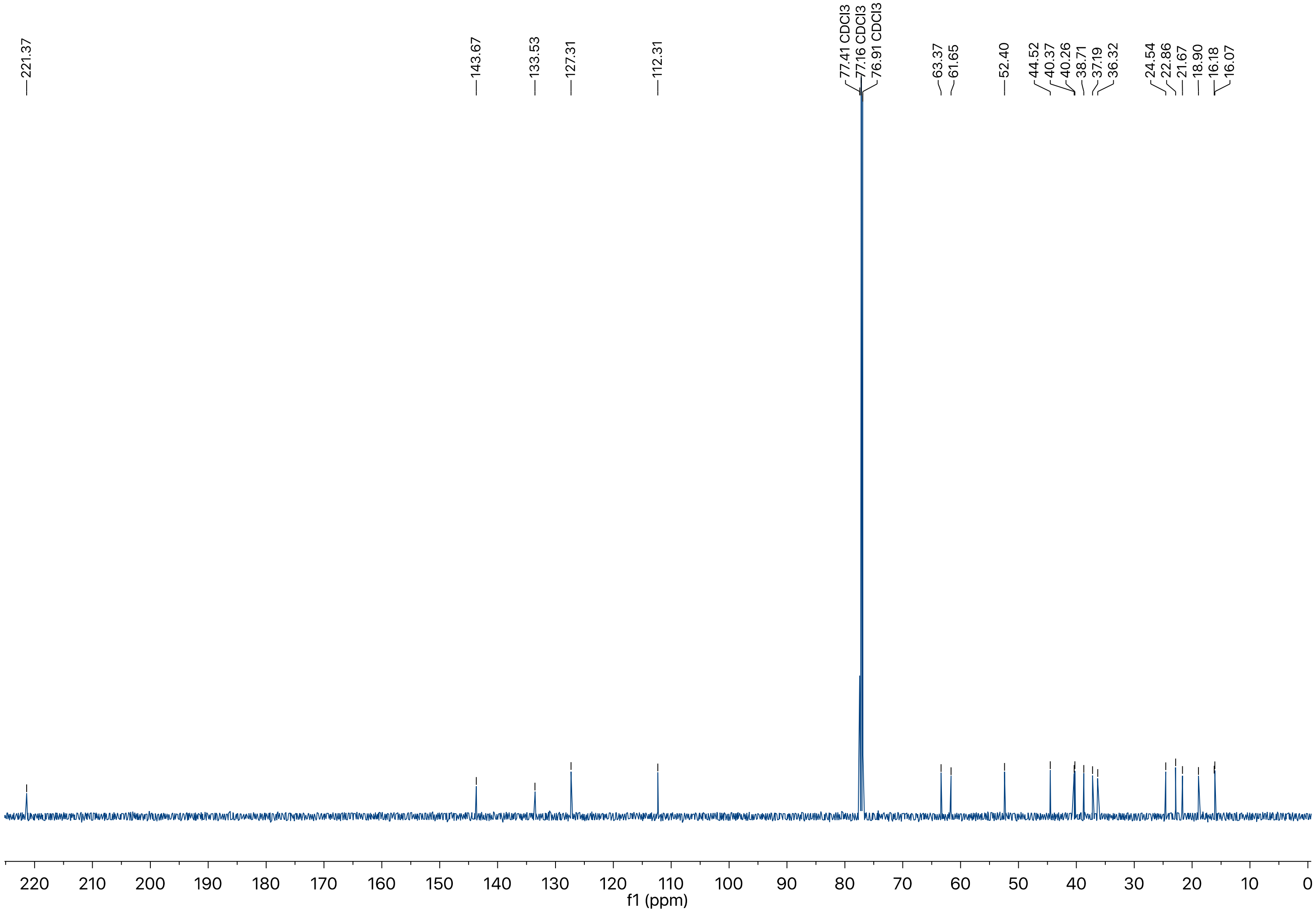

Figure S16. ^{1}H NMR spectrum of (1*R*,3*E*,7*E*,11*S*,12*S*)-14-oxo-3,7,18-dolabellatriene (**5**) (500 MHz, $CDCl_3$)

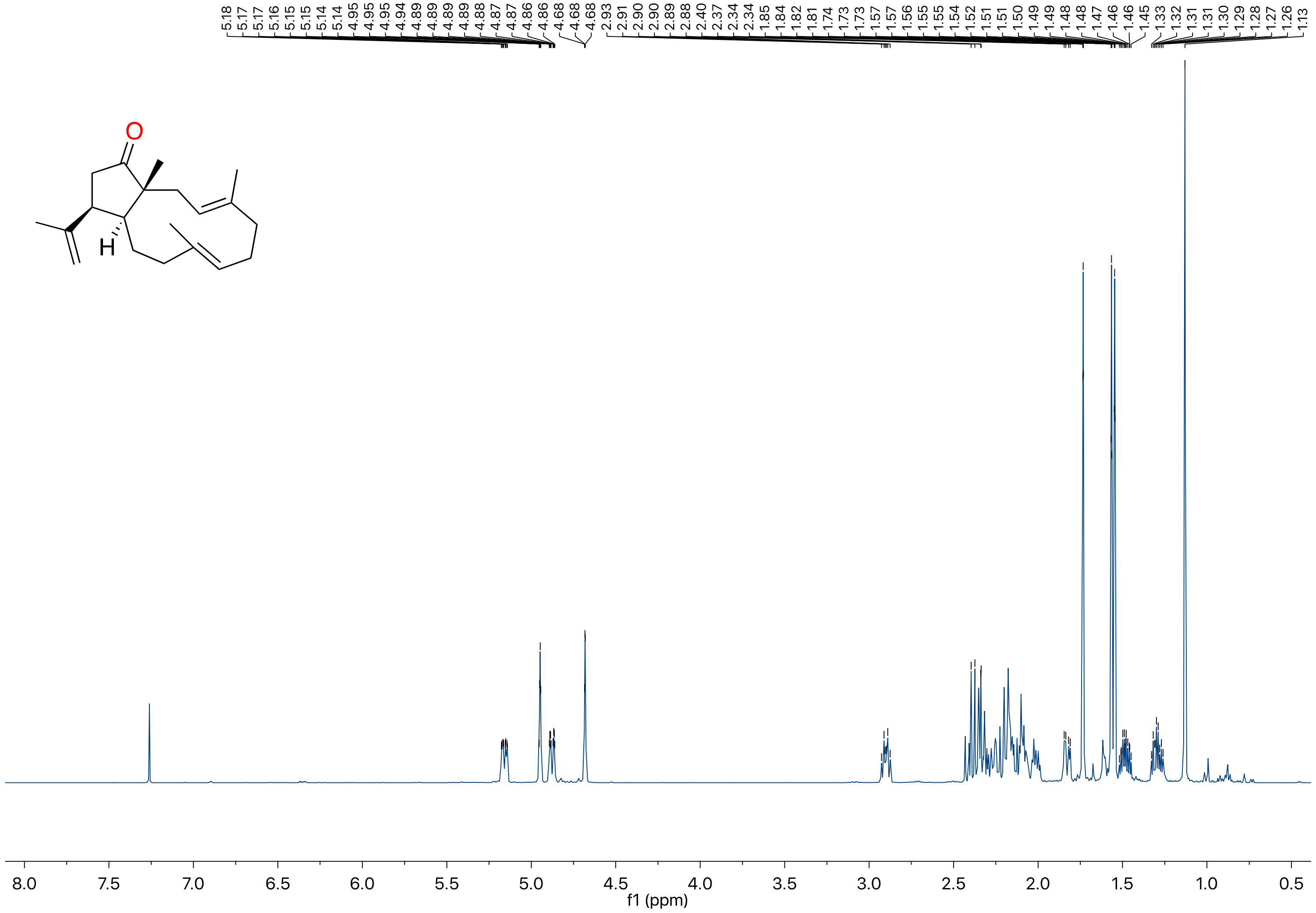

Figure S17. ^{13}C NMR spectrum of (1*R*,3*E*,7*E*,11*S*,12*S*)-14-oxo-3,7,18-dolabellatriene (**5**) (125 MHz, $CDCl_3$)

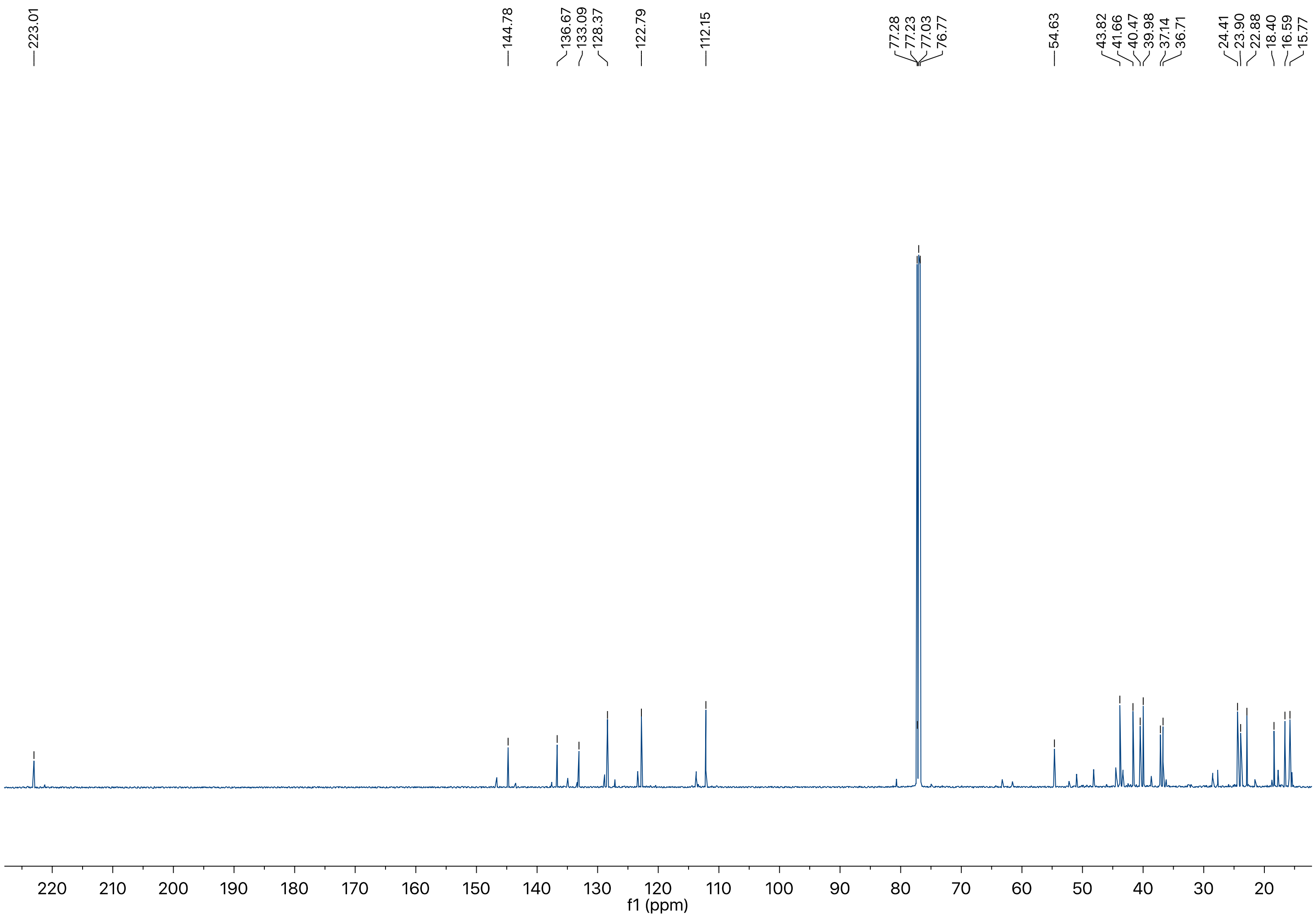

Figure S18. ^{1}H NMR spectrum of (1*R*,3*Z*,7*E*,11*S*,12*S*)-14-oxo-3,7,18-dolabellatriene (**6**) (500 MHz, $CDCl_3$)

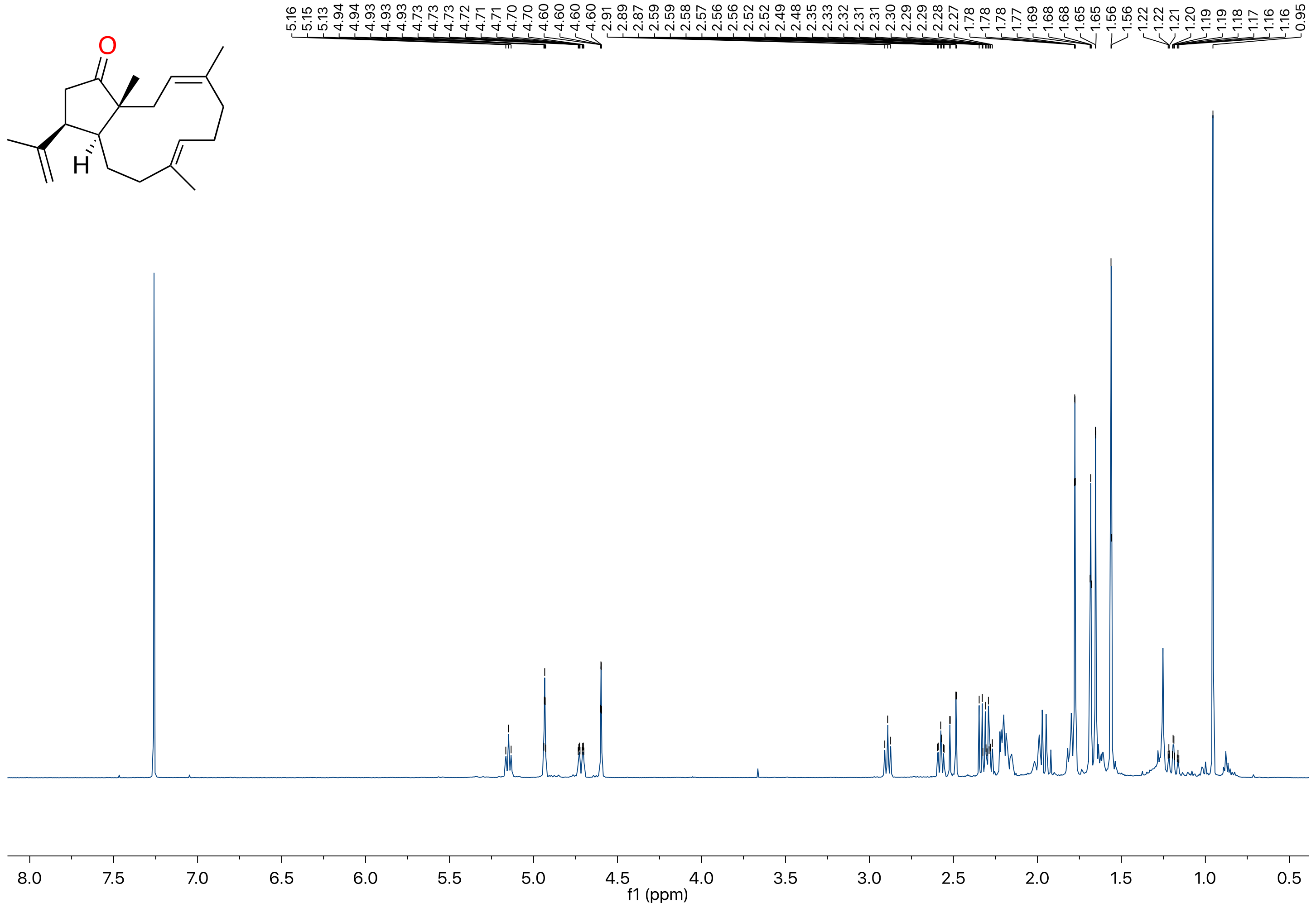

Figure S19. ^{13}C NMR spectrum of (1*R*,3*Z*,7*E*,11*S*,12*S*)-14-oxo-3,7,18-dolabellatriene (**6**) (125 MHz, $CDCl_3$)

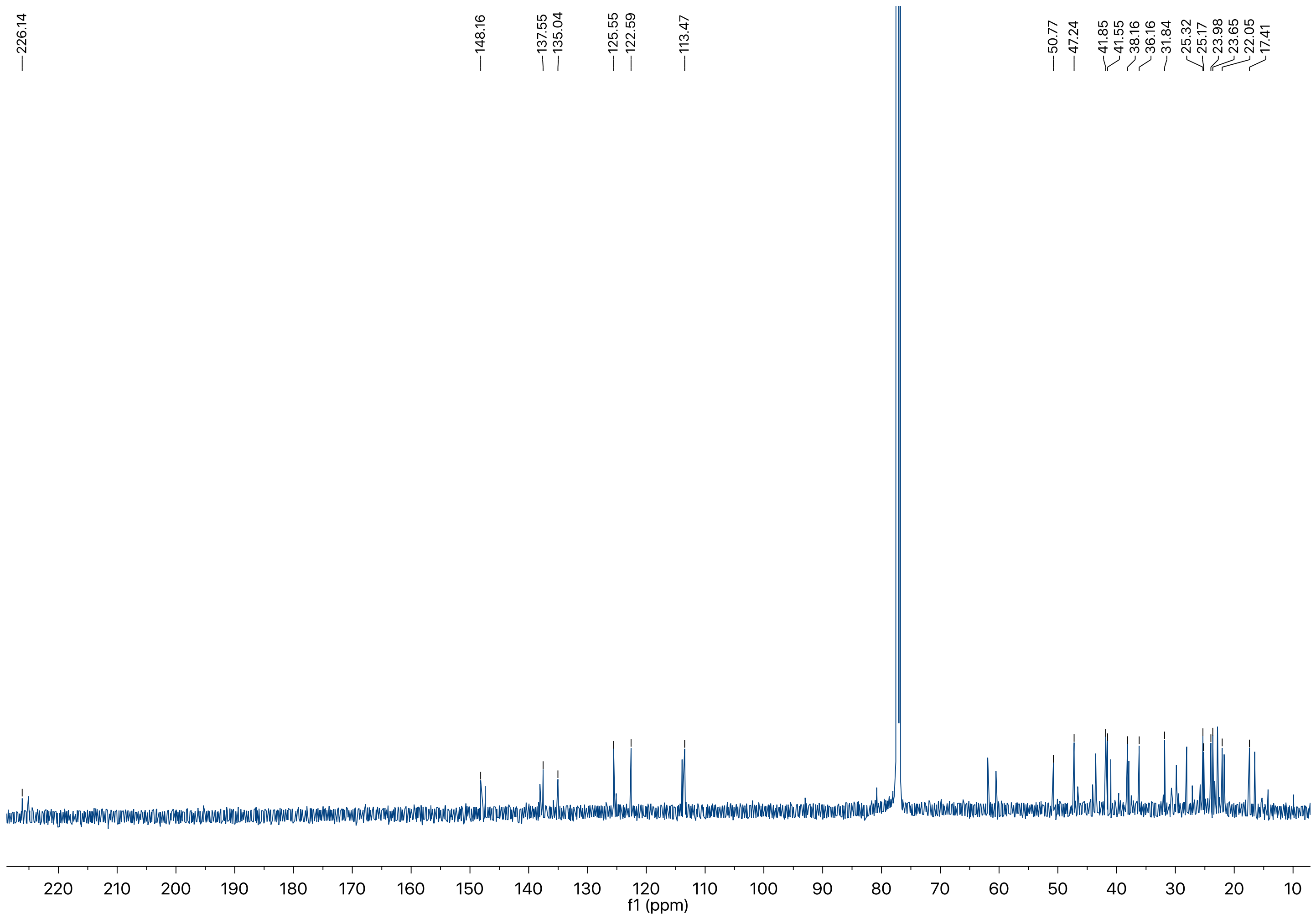

Supplement: Supplementary file 1 [file marinedrugs-17-00192-s001.pdf]
